# A Comprehensive Mouse Transcriptomic BodyMap across 17 Tissues by RNA-seq

**DOI:** 10.1038/s41598-017-04520-z

**Published:** 2017-06-23

**Authors:** Bin Li, Tao Qing, Jinhang Zhu, Zhuo Wen, Ying Yu, Ryutaro Fukumura, Yuanting Zheng, Yoichi Gondo, Leming Shi

**Affiliations:** 1 Center for Pharmacogenomics, School of Pharmacy, and State Key Laboratory of Genetic Engineering, School of Life Sciences and Shanghai Cancer Hospital/Cancer Institute, Fudan University, Shanghai, 200438 China; 2 Collaborative Innovation Center for Genetics and Development, Fudan University, Shanghai, 200438 China; 3 College of Chemistry, Sichuan University, Chengdu, 610064 China; 4 Mutagenesis and Genomics Team, RIKEN BioResource Center, Tsukuba, Ibaraki 305-0074 Japan

## Abstract

The mouse has been widely used as a model organism for studying human diseases and for evaluating drug safety and efficacy. Many diseases and drug effects exhibit tissue specificity that may be reflected by tissue-specific gene-expression profiles. Here we construct a comprehensive mouse transcriptomic BodyMap across 17 tissues of six-weeks old C57BL/6JJcl mice using RNA-seq. We find different expression patterns between protein-coding and non-coding genes. Liver expressed the least complex transcriptomes, that is, the smallest number of genes detected in liver across all 17 tissues, whereas testis and ovary harbor more complex transcriptomes than other tissues. We report a comprehensive list of tissue-specific genes across 17 tissues, along with a list of 4,781 housekeeping genes in mouse. In addition, we propose a list of 27 consistently and highly expressed genes that can be used as reference controls in expression-profiling analysis. Our study provides a unique resource of mouse gene-expression profiles, which is helpful for further biomedical research.

## Introduction

The mouse shares more than 15,000 protein-coding genes with humans^1, 2^ and suffers from most diseases of mankind, making it a widely used model organism for biomedical research and for the study of human diseases. Gene expression, primarily measured by mRNA levels, varies across different tissues, cell types and developmental times^3^, and also can be an indicator of different cellular states^4^ (e.g. health versus disease). With the advances of next-generation sequencing technologies over the past several years, RNA sequencing (RNA-seq) can provide the expression profiles of the entire transcriptome of a sample at a single-nucleotide resolution without knowing the genetic sequence *a priori* and with unprecedented high throughput^5–7^. Great efforts have been made for investigating the RNA-seq transcriptome analyses across tissues and individuals of human^8, 9^ and rat^10^. Such efforts are needed for mouse as well.

With the endeavor of the FANTOM consortium^11–13^, the mouse genome has been annotated quite well and is helpful for gene-expression profiling studies of mouse. In addition, the mouse ENCODE project—part of the ENCODE program—mainly focuses on the catalog of functional elements, e.g., transcription capping sites, in the human and mouse genomes as well as the divergence and conservation between these two genomes^2, 14, 15^. Several databases have been constructed for gene-expression profiles, such as the BodyMap^16^ based on 3′-ESTs (Expressed Sequence Tags) for both mouse and human, the EMAGE database of *in situ* gene-expression data in the mouse embryo^17^, and TiSGeD for tissue-specific genes in mouse, rat, and human across different tissues using microarray data sets^18^. Previous studies demonstrated that some diseases are highly tissue-specific^19–21^ and may be reflected by genes expressed specifically in a given tissue. Therefore, it is of high value to construct a standardized comprehensive transcriptomic BodyMap across different tissues of mouse in a defined genetic background under a controlled environment for understanding the transcriptional machinery of the cell and building suitable mouse models to study human diseases.

In our study, we use RNA-seq to build a comprehensive transcriptomic profiling data set across 17 tissues at sexually matured developmental stage in both sexes of C57BL/6JJcl mice. We found that the protein-coding genes (PCGs) and non-coding genes manifested quite differently in expression patterns. We also identified tissue-specific genes that were highly associated with the particular biological processes or development of a specific tissue. In addition, 4,781 candidates were identified as mouse housekeeping genes among which a list of 27 genes that are expressed highly consistently and highly across all tissues are proposed as an internal control set for experiments in various conditions as well as for normalizing gene-expression data. Our study and data set should provide a fundamental resource for the study of mammalian gene-expression profiles encompassing human disease modeling.

## Results

### Study design

To construct a comprehensive transcriptomic BodyMap of mouse, we generated an RNA-seq data set using a total of 72 samples isolated from 2 female and 2 male C57BL/6JJcl mice of 6-weeks old. Fourteen non-sexual tissues (adrenal gland, bone marrow, brain, forestomach, heart, kidney, liver, large intestine, lung, muscle, small intestine, spleen, stomach, and thymus) and two sexual tissues (ovary and uterus for females, or testis and vesicular gland for males) with two replicate samples of brain, liver, kidney and testis for each mouse and one sample of the other tissues per mouse were subjected to the RNA-seq analysis as follows (Table 1). Total RNA was extracted from each tissue by using the miRNeasy Mini Kit (Qiagen) according to the manufacturer’s protocol, and the RNA quality was evaluated with an Agilent Bioanalyzer. Then we used Illumina HiScan platform to sequence each cDNA library except four kidney and two testis samples for some reasons (Supplementary Table S1). On average, about 10 million 101 * 101 bp pair-ends reads were generated per sample, yielding a total of 1.4 billion reads for the study. Based on the quality reports of the raw data generated by fastQC^22^, mainly per base GC contents and per base sequence quality, we performed sequence trimming using Trimmomatic^23^ under default parameter settings. On average, 1.05 million reads (~10.6%) were eliminated from each sample (Supplementary Table S2). All subsequent analyses were based on the remaining reads after trimming.

**Table 1:** Mice and tissue sampling.

Individual ID		**Mouse 1**	**Mouse 2**	Mouse 3	Mouse 4
Sex		**Male**	**Male**	Female	Female
Birth Date		31-Mar-08	31-Mar-08	31-Mar-08	31-Mar-08
Sampling Date		13-May-08	13-May-08	13-May-08	13-May-08
Type of Tissue	Abbreviation	# of Samples	# of Samples	# of Samples	# of Samples
Brain	Br	2*	2*	2*	2*
Liver	Li	2*	2*	2*	2*
Kidney	Ki	2*	2*	2*	2*
Adrenal gland	Ag	1	1	1	1
Spleen	Sp	1	1	1	1
Lung	Lu	1	1	1	1
Heart	He	1	1	1	1
Thymus	Th	1	1	1	1
Ovary	Ov			1**	1**
Testis	Te	2*	2*		
Bone marrow***	Bm	1	1	1	1
Stomach	St	1	1	1	1
Forestomach	Fs	1	1	1	1
Small intestine	Sin	1	1	1	1
Large intestine	Lin	1	1	1	1
Muscle	Mu	1	1	1	1
Uterus	Ut			1	1
Vesicular gland	Vg	1	1		

*Two independent samples from Br, Li, Ki & Te were collected from each mouse. **The pair of Ov per mouse was pooled into one sample. ***The data of Bm were eliminated from the detailed analyses as shown in the text.

### Overview of the landscape of the mouse transcriptome

**Figure 1:**
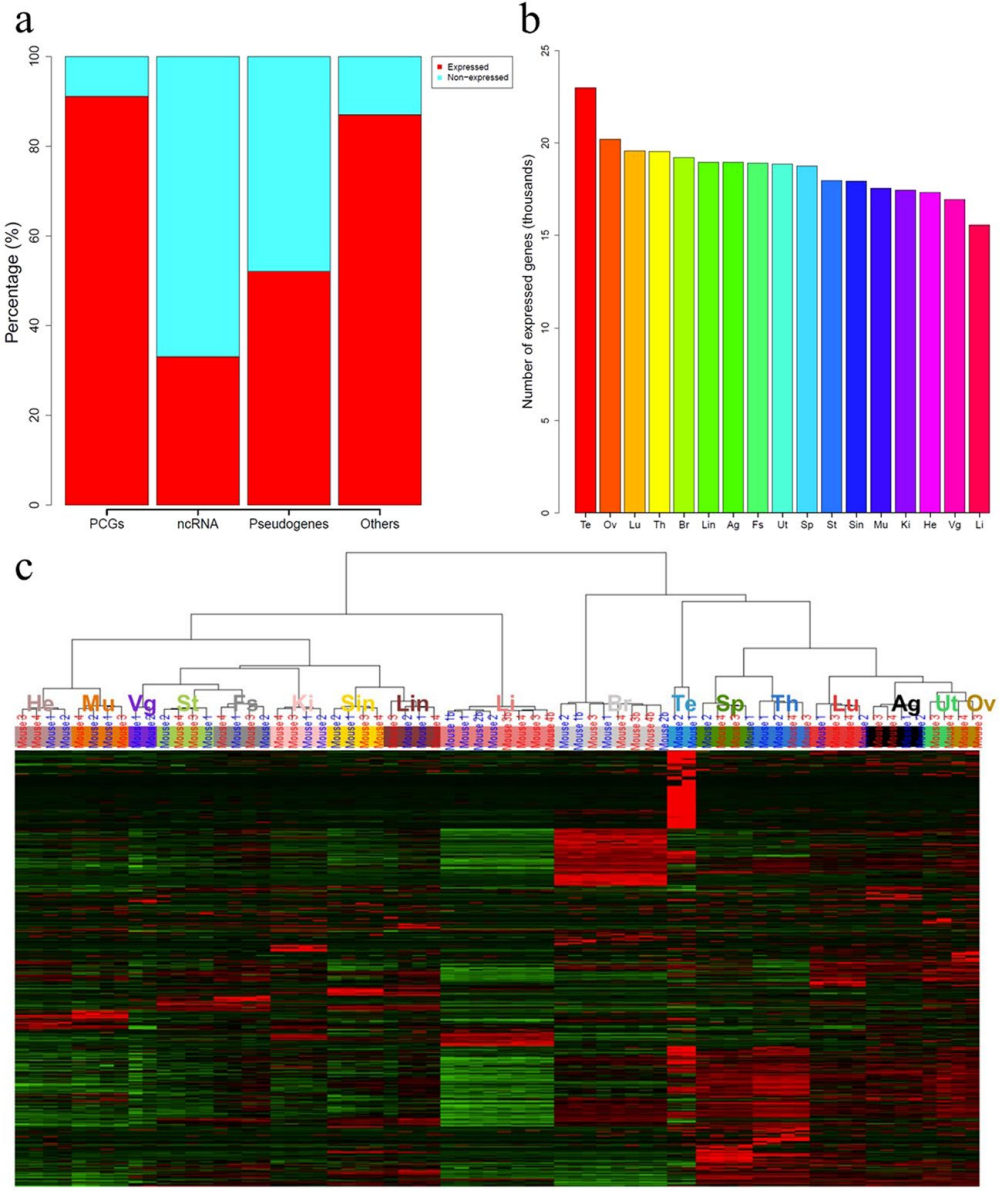
Landscape of the mouse transcriptome. (**a**) Percentage of expressed (red, FPKM > = 0.1 in at least one sample) and non-expressed (blue, FPKM < 0.1 across all samples) genes by different gene types. PCGs: protein-coding genes; ncRNA: non-coding RNA genes; Others: other genes except PCGs, ncRNA, and pseudogenes. (**b**) Number of expressed (FPKM > 0.1) genes by tissues. Numbers were averaged over all biological samples for a given tissue. (**c**) Hierarchical clustering analysis of gene expression profiles of 68 samples with 31,687 genes. Each column indicated a sample, whereas each row indicated a gene. Each tissue symbol was shown upon the color bar for each cluster. The mouse information where each sample came from was also labeled upon the color bar. Mouse 1 and Mouse 2 were two male mice (in blue text), while Mouse 3 and Mouse 4 were two female mice (in red text) as shown in Table 1.

With all the biological samples combined, a raw data matrix of fragments per kilobase of exon per million mapped fragments (FPKM) values consisting of 43,628 genes annotated in the Ensembl GRCm38 mouse reference genome across 72 samples was generated at first. It must be noted that the hierarchical cluster analysis of all the 72 samples (Supplementary Fig. S1) showed that one of the bone marrow samples was not clustered together with the other three bone marrow samples, indicating that there may be some problems such as a mislabeling with this sample. Since we were unable to trace down what exactly happened to this sample during the experiment, we removed all the four bone marrow samples hereafter, resulting in 68 remaining samples from 17 tissues for this study to enhance the reliability of our analyses. Of all the 43,628 genes, 22,196, 8,936, 8,060, and 4,436 belong to protein coding genes (PCGs), noncoding RNA (ncRNA), pseudogenes, and other genes (except the above three types), respectively. As reported in a previous study^8^, when the threshold of expression was set to FPKM > = 0.1 in at least one sample for a given gene, most PCGs (20,261/22,196, 91%) were expressed in some mouse tissues, whereas only a much smaller portion of the ncRNA genes (2,939/8,936, 33%) were expressed. That is, most ncRNA genes were typically tissue-specific or not expressed in any of the tissues studied. The percentages of expressed and non-expressed pseudogenes were almost even (52% vs 48%). Interestingly, the expression of the remaining 4,436 genes belonging to other categories showed a similar pattern as the PCGs, i.e., most of them were expressed, implying that these genes may play a role like PCGs in the activities of cells (Fig. 1a). This complementary expression pattern between PCGs and ncRNA was also observed under different thresholds (0.1 and 1) when considering the number of expressed genes given the number of tissues (Supplementary Fig. S2). Our results were similar to those found by the Genotype-Tissue Expression (GTEx) project^8^. However, the percentage of ncRNAs detected in at least one sample in our study was lower than that of the GTEx project (33% compared to 71%). It may be partly due to much fewer samples and tissues in our study compared to the GTEx project.

The number of genes expressed in a given tissue ranged from 15,578 to 22,295. On average 18,634 (42.7%) of the 43,628 genes were defined as expressed (FPKM > = 0.1) per tissue. Differences among tissues in the numbers of genes expressed were observed. The testis expressed the most genes, followed by ovary and lung, which was consistent with findings in human^9^ and rat^10^, whereas the liver expressed the least number of genes (Fig. 1b). In addition, liver, heart, and kidney expressed relatively fewer genes than other tissues in all the three species. It proved in a way that the same tissues from different species exhibit high similarity. We also identified 11,017 genes and 10,926 isoforms ubiquitously expressed (FPKM > = 0.1 across all the 68 samples) which should play a vital role in basic biological functions of cells.

To analyze the gene expression profiles in a logarithmic form, genes with FPKM values of zero across all 68 samples were removed, and the value of 1 was added to each FPKM value before log2 transformation. Thus, a final data matrix of 31,687 genes and 68 samples was generated for subsequent analyses. Of the remaining 31,687 genes, 20,387 and 2,985 belong to PCGs and ncRNA, respectively. Hierarchical cluster analysis (Fig. 1c) as well as principal component analysis (Supplementary Fig. S3) of all the 68 samples demonstrated that different tissue types exhibited unique gene-expression profiles. Morphologically or functionally similar tissue pairs, such as heart versus muscle, stomach versus forestomach, and ovary versus uterus, were clustered more closely to each other, indicating that these tissue pairs shared a more consistent expression profile than other tissues. Both brain and liver showed relatively large differences in gene-expression profiles with the other tissues (Supplementary Fig. S3).

### Tissue-dependent differentially expressed genes

**Figure 2:**
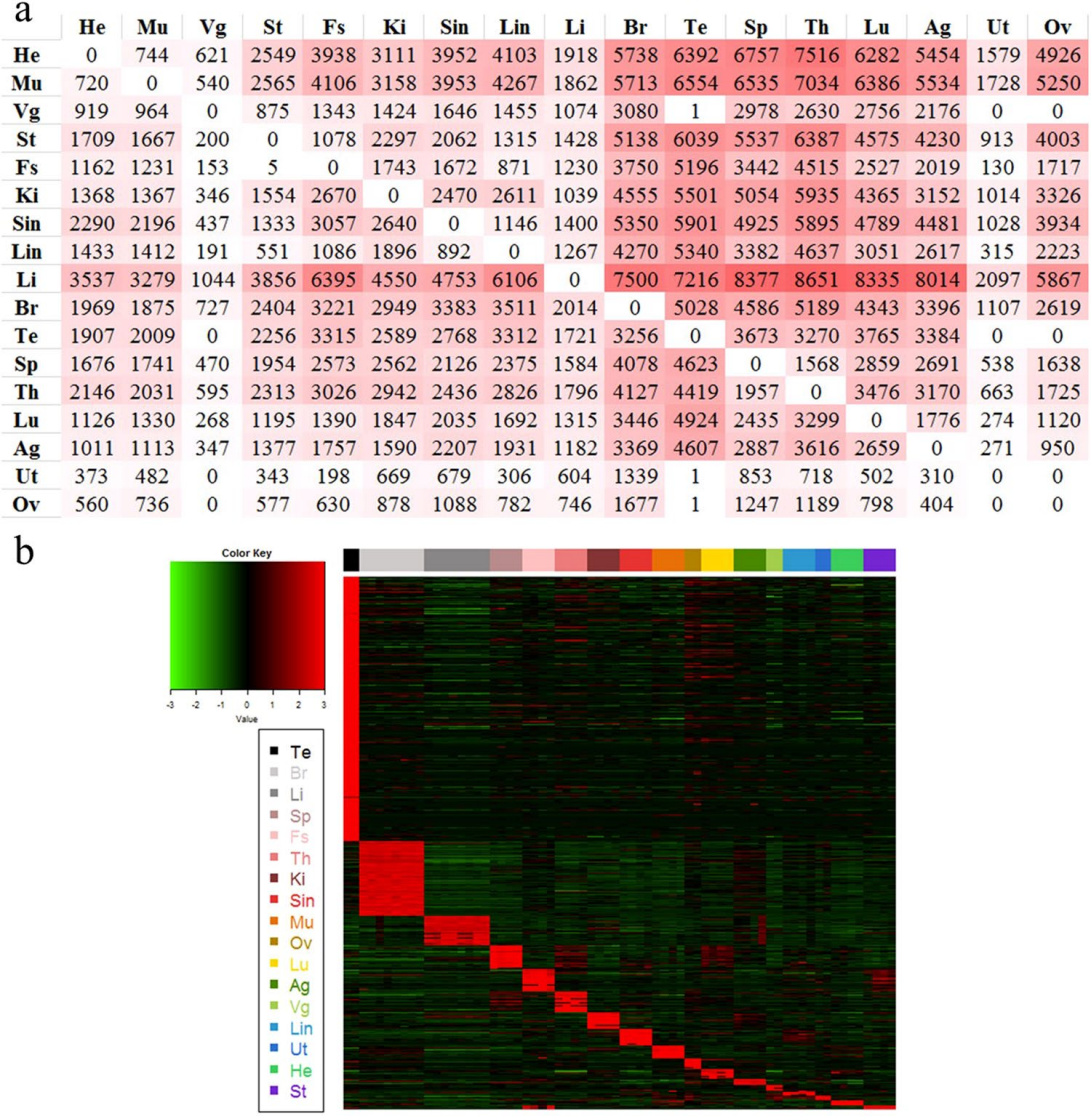
Tissue-dependent differentially expressed genes. (**a**) Number of differentially expressed genes (FDR < 0.05, fold change >2 or <0.5) for each pairwise organs. For each tissue in each column, the figures indicated the numbers of down-regulated genes comparing this tissue to the other tissues. For each tissue in each row, the figures indicated the numbers of up-regulated genes comparing this tissue to the other tissues. The tissues are in the same order as in Fig. 1c. (**b**) Expression profiles of 5,035 tissue-specific genes across 68 samples were arranged by tissue types (in decreasing order based on the number of organ-enriched genes). Testis expressed the most tissue-specific genes among all tissues.

We conducted a pairwise comparison using a t-test (FDR < 0.05, fold change (FC) > = 2 or <= 0.5) to identify differentially expressed genes (DEGs) between any two tissues (for all of the 17 tissues). The numbers of DEGs were largely dependent on the two types of tissues being compared. DEGs in the heart, liver, and muscle were generally under-expressed compared with other tissues, whereas DEGs were generally over-expressed compared with other tissues in adrenal gland, brain, lung, spleen, testis, and thymus (Fig. 2a). Our results were similar to a previous study on the rat transcriptome^10^. We also conducted separate pairwise comparison analyses for PCGs or ncRNA only (Supplementary Fig. S4). It was found that the PCGs had a quite similar pattern with the whole set of genes, whereas the ncRNA genes showed less divergence between tissues. Only brain, testis, and thymus had relatively high number of over-expressed ncRNA genes compared with other tissues.

We also identified tissue-specific genes that are defined as those with more than four-fold higher expression in a given tissue over any other tissues. Totally, we identified 5,035 tissue-specific genes, with a relatively small number for each tissue except testis and brain (Fig. 2b). The number of tissue-specific genes varied from tissue to tissue. Testis expressed the most tissue-specific genes (2,496), closely followed by brain (708) and liver (280). The numbers of tissue-specific genes identified in the other ten tissues ranged from 41 to 223 (Supplementary Fig. S5).

DAVID online tools^24^ were used to reveal the biological meaning behind these tissue-specific genes. Generally, these genes were highly correlated with the particular biological processes or the development of a specific tissue (Supplementary Table S3). For example, the brain-specific genes were mainly associated with neural processes such as the transmission of nerve impulse and synaptic transmission as well as the KEGG^25^ pathway of neuroactive ligand-receptor interaction. The heart-specific and muscle-specific genes were involved in the biological processes of heart development and muscle tissue development, respectively. Furthermore, processes involved in the metabolism and acute inflammatory response were identified in liver-specific genes, whereas processes related to transport were enriched with kidney-specific genes. Genes specifically expressed in the four sexual tissues were mainly enriched in gene ontology categories associated with sex-related biological processes such as sexual reproduction, reproductive structure development, gamete generation, embryonic limb morphogenesis, and mating plug formation.

### Sex-dominated genes expressed in non-sexual tissues

We also conducted comparisons between female and male mice to identify sex-dominated genes for the 13 non-sexual tissues (Supplementary Fig. S6). Overall, we identified 1,682 female-dominated genes (genes with expression level in female more than two-fold higher than in male) and 2,377 male-dominated genes (genes with expression level in male more than two-fold higher than in female) across all 13 tissues. Some tissues such as heart and spleen showed relatively large difference between female and male in the number of sex-dominated genes. Meanwhile, different patterns were observed in the differences between the numbers of female and male dominated genes within a specific tissue across all 13 tissues. Some tissues expressed an equivalent number of sex-dominated genes for both sexes (such as adrenal gland, brain, kidney, liver, and thymus), whereas forestomach, heart, large intestine, lung, muscle, and spleen expressed more male-dominated genes, and small intestine and stomach expressed more female-dominated genes (Supplementary Fig. S7). Though each tissue had only two replicates (except brain and liver which had four replicates) for each sex, our results can somehow show the sex differences in non-sexual tissues.

### Identification of housekeeping genes

Housekeeping genes are typically constitutive genes that are required for the maintenance of basic cellular functions, and are expressed in all cells of an organism under different conditions^26–28^. Thus, housekeeping genes are widely used as internal controls for experiments as well as for normalizing gene expression data^29–31^. To identify housekeeping genes in mouse, we used similar criteria as a previous study^32^ (see Methods).

**Figure 3:**
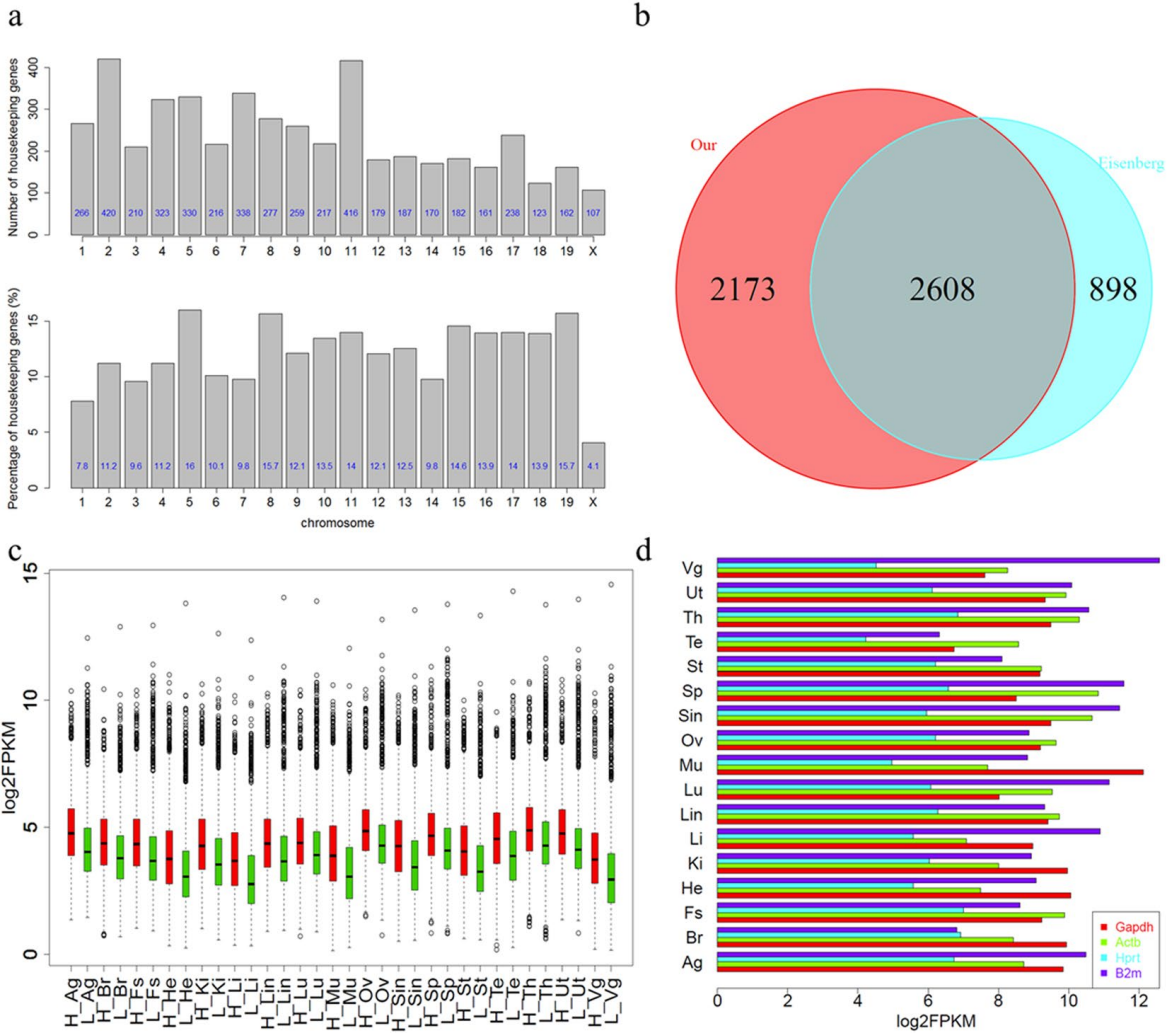
4,781 genes were identified as mouse candidate housekeeping genes. (**a**) Chromosome distribution of the 4,781 housekeeping genes. The exact number for each chromosome was highlighted in blue figure. Upper panel: the absolute number of housekeeping genes identified on each chromosome; lower panel: percentage of housekeeping genes on each chromosome (divided by the total number of genes on each chromosome). (**b**) 2,608 genes were both identified as housekeeping genes in mouse (our study, red circle) and human (Eisenberg’s study, blue circle). (**c**) Expression profiles of the 4,781 genes by groups. Red: genes also identified as housekeeping genes in human; Green: genes not identified in human. (**d**) Expression of four well-known housekeeping genes (Gapdh, Actb, Hprt, and B2m). Only Hprt was found in our list of housekeeping genes.

Hence, we identified 4,781 candidate housekeeping genes satisfying the above criteria. The vast majority of these housekeeping genes (4,662) belonged to protein-coding genes, whereas the rest were classified as lncRNA (30), processed pseudogene (24), processed transcript (23), antisense (23), and so on. The distribution of these genes varied across chromosomes (Fig. 3a). Chromosome 2 harbored the most housekeeping genes (420), closely followed by chromosome 11 (416), while chromosome X had the least housekeeping genes (107) without taking account for chromosome Y because no housekeeping genes were found on chromosome Y. However, after normalizing the data by the number of all the genes on each chromosome, we found chromosome 5 had the highest percentage of housekeeping genes, followed by chromosome 8 and 19. To assess the reliability of our results, we compared our gene list to a list of 3,804 human housekeeping genes^32^ based on human and mouse homologous genes^33^. Of the 3,506 human housekeeping genes having a homology to mouse genes, 2,608 (74.4%) were found in our candidate mouse housekeeping genes. Conversely, among the 4,781 candidate mouse housekeeping genes from our study having a homology to human genes, 2,608 (54.5%) were also found in the list of human housekeeping genes (Fig. 3b). The discrepancy may be due to the true differences between species or the types of tissues included in the respective studies.

According to the comparison results, we divided the candidate mouse housekeeping genes into two groups: genes with high confidence (the 2,608 genes that are in common with the human housekeeping genes) and relatively low confidence (the remaining 2,173 genes). Interestingly, the expression level of the former group was always higher than the latter one within the corresponding tissue (Fig. 3c). The higher measurement variability for the relatively lower expression genes might account for the differences between human and mouse.

As expected, the housekeeping genes are enriched in biological processes or pathways related to basic cellular activities, such as RNA processing, translation, protein localization, protein transport, and so on. Separate analyses of the two groups of housekeeping genes demonstrated that they shared 186 and 12 significantly enriched (p-value < 0.05) GO terms and KEGG pathways (Supplementary Fig. S8) mostly associated with fundamental activities of cells, respectively.

Furthermore, we particularly focused on the expression profiles of four well-known housekeeping genes, Gapdh, Actb, Hprt, and B2m, which were widely used as internal references to normalize RNA expression levels (Fig. 3d). However, only Hprt was found in our list of candidate housekeeping genes, because the other three genes were highly variable among tissues and exhibited more than 41, 13, and 77 fold maximal variability for Gapdh, Actb, and B2m, respectively. Our observations are consistent with results from previous studies^32, 34^, indicating that Gapdh, Actb, and B2m are probably not suitable for the reference purposes.

Consequently, we proposed a list of 27 genes that are consistently and highly expressed across all tissues included in this study (Table 2). The expression profiles of these 27 genes (Supplementary Table S5) are much more consistent than currently commonly used housekeeping genes such as Gapdh, Actb, and B2m. Thus, we recommend using the 27 candidate genes as the reference, e.g., for the normalization of the gene-expression studies.

**Table 2:** Twenty-seven (27) consistently and highly expressed mouse housekeeping genes.

No.	Symbol	Definition	Type	Chr
1	Arf1	ADP-ribosylation factor 1	protein_coding	chr11
2	Cox7a2l	cytochrome c oxidase subunit VIIa polypeptide 2-like	protein_coding	chr17
3	D8Ertd738e	DNA segment, Chr 8, ERATO Doi 738, expressed	protein_coding	chr8
4	Eif1	eukaryotic translation initiation factor 1	protein_coding	chr11
5	Eif4g2	eukaryotic translation initiation factor 4, gamma 2	protein_coding	chr7
6	Eif5a	eukaryotic translation initiation factor 5A	protein_coding	chr11
7	Gabarap	gamma-aminobutyric acid receptor associated protein	protein_coding	chr11
8	Grcc10	gene rich cluster, C10 gene	protein_coding	chr6
9	Myeov2	myeloma overexpressed 2	protein_coding	chr1
10	Ndufa2	NADH dehydrogenase (ubiquinone) 1 alpha subcomplex, 2	protein_coding	chr18
11	Ndufa7	NADH dehydrogenase (ubiquinone) 1 alpha subcomplex, 7	protein_coding	chr17
12	Nedd8	neural precursor cell expressed, developmentally down-regulated gene 8	protein_coding	chr14
13	Oaz1	ornithine decarboxylase antizyme 1	protein_coding	chr10
14	Pomp	proteasome maturation protein	protein_coding	chr5
15	Psma2	proteasome (prosome, macropain) subunit, alpha type 2	protein_coding	chr13
16	Psma3	proteasome (prosome, macropain) subunit, alpha type 3	protein_coding	chr12
17	Psma4	proteasome (prosome, macropain) subunit, alpha type 4	protein_coding	chr9
18	Psmb1	proteasome (prosome, macropain) subunit, beta type 1	protein_coding	chr17
19	Psmb2	proteasome (prosome, macropain) subunit, beta type 2	protein_coding	chr4
20	Psmb3	proteasome (prosome, macropain) subunit, beta type 3	protein_coding	chr11
21	Psmb4	proteasome (prosome, macropain) subunit, beta type 4	protein_coding	chr3
22	Psmb6	proteasome (prosome, macropain) subunit, beta type 6	protein_coding	chr11
23	Psmb7	proteasome (prosome, macropain) subunit, beta type 7	protein_coding	chr2
24	Rab1b	RAB1B, member RAS oncogene family	protein_coding	chr19
25	Saraf	store-operated calcium entry-associated regulatory factor	protein_coding	chr8
26	Ubl5	ubiquitin-like 5	protein_coding	chr9
27	Vcp	valosin containing protein	protein_coding	chr4

## Discussion

The characterization of gene expression is a powerful way to identify the differences of the transcriptional machinery between tissues and diverse status of cells. Here we constructed a comprehensive transcriptome map of the standard mouse strain, C57BL/6JJcl, which had branched in 1989 from the original C57BL/6J, by describing the gene-expression profiles across 17 tissues from both sexes. Unlike human studies, mouse inbred strains provide reproducible experimental materials with a well-defined genetic background for biological studies. The C57BL6/J inbred strain is the source of the mouse genome reference^35^, current version GRCm38.4 (http://www.ncbi.nlm.nih.gov/projects/genome/assembly/grc/mouse/). The addition of the transcriptomic BodyMap described here of C57BL/6JJcl, which has the basically the same genetic background of C57BL/6J, should make the mouse a more useful resource as a model organism.

We found different expression patterns between protein-coding and non-coding genes as observed by the GTEx study^8^. Liver and adrenal gland expressed the least complex transcriptome, in that they expressed the minimum number of genes, whereas testis and ovary harbored more complex transcriptome than other tissues. We also observed about 11,000 genes ubiquitously expressed across all 17 tissues examined in our study. This number is much higher than the ~1,800 shared genes detected by microarray^36^, but was quite equivalent with the ~10,000 shared genes found by reassociation kinetics^37^. However, it should be noted that the microarray study^36^ had included 55 mouse tissues of different developmental stages.

Genes specifically expressed in a particular tissue were traditionally supposed to be associated with tissue-specific functions. For example, genes specifically expressed in brain were mainly involved in neural processes such as synaptic transmission. In addition, some diseases were highly tissue-specific, which may be highly correlated with tissue-specific gene expression^19–21^. In our study, we proposed a relatively comprehensive tissue-specific gene list across 17 tissues. We found that testis expressed the largest number of tissue-specific genes, whereas the stomach expressed the lowest number of tissue-specific genes. Functional annotation of these genes demonstrated that they were involved in tissue-specific biological processes or tissue development. Thus, our data set should also provide a rich resource for research toward tissue-specific functions.

Housekeeping genes were traditionally viewed as essential for the maintenance of basic cellular functions and were ubiquitously and uniformly expressed in different biological conditions^32^. Although several efforts of identifying housekeeping genes have been made for human^32, 38, 39^, such efforts were much less advanced for mouse. Here by analyzing the gene-expression patterns using RNA-seq across 17 diverse mouse tissues, we identified a comprehensive list of 4,781 mouse housekeeping genes. The set of housekeeping genes described here showed a good agreement with that of human housekeeping genes previously reported by Eisenberg and Levanon^32^. That is, 54.5% of our mouse housekeeping genes were included in the set of 3,506 human housekeeping genes, and 74.4% of the 3,506 human housekeeping genes were also found in our list of the mouse housekeeping genes. Furthermore, we proposed a list of 27 genes that are consistently and highly expressed across all mouse tissues for potential use as internal references in gene-expression studies.

The FANTOM consortium conducted extensive studies on the functional annotation of mammalian genome since 2001 and provides valuable data resources for biomedical research. In FANTOM1^11^ and FANTOM2^12^, they constructed full-length cDNA libraries from various mouse organs and developmental stages, followed by the determination of their sequences by Sanger method. Then, they have started series of quantitative transcriptome studies mainly focusing on the use of capping sites by using Cap Analysis of Gene Expression (CAGE)^40^. More recently, scientists in FANTOM5 produced a comprehensive overview of promoter-level mammalian expression atlas in human and mouse primary cells, cell lines and tissues by using CAGE with the Helicos platform. In general, CAGE conducts a deep-tag sequencing of short 5′ ends of transcripts, while RNA-seq identifies shotgun sequencing of entire RNA sequences. CAGE is surely the first choice for annotating 5′ ends of transcripts (mainly related to transcription start sites (TSS) including promoter and enhancer) while RNA-seq is better at annotating transcript structure^41^. Although both technologies have strengths and weaknesses, scientists may use them as complementary technologies to reveal and refine the complexity and estimating the expression levels of individual genes^42^.

Though our data should serve as a valuable and complementary resource for biomedical research together with FANTOM5 data generated by CAGE, we must pay special attention to the basic features (such as age, sex, and so on) of the two datasets as well as batch effects between them. Takao & Miyakawa^43^ and Seok *et al*.^44^ drew diametrically opposite conclusions using the same datasets. It turned out Seok *et al*.^44^ used inappropriate methods for data analysis and made comparisons between incomparable datasets misleading to biased conclusions. Contrary, Takao & Miyakawa^44^ proposed proper adjustments for data analysis by appropriately integrating datasets among different studies, platforms, and species. Similar features should be chosen and batch effects should be eliminated before making any comparisons between our dataset and those from FANTOM5. Otherwise we may draw incorrect conclusions.

In summary, we have generated an extensive gene-expression data set consisting of 17 solid mouse tissues for both sexes. Our study has provided a unique resource of mouse gene-expression profiles and is helpful for further biological function research. The well-defined genome reference sequence and comprehensive transcriptomic BodyMap in the standardized inbred mouse strain C57BL/6JJcl should facilitate the understanding of human diseases and constructing applicable mouse models in biomedical research.

## Methods

### Mice and tissue sampling

Four six-weeks-old C57BL/6JJcl mice (two males; Mouse-1 and Mouse-2, and two females; Mouse-3 and Mouse-4) were purchased from CREA Japan and subjected to transcriptomic analysis in this study. Notably, C57BL/6JJcl branched from the original C57BL/6J in 1989; therefore, their genetic backgrounds are basically the same. From each mouse, 14 non-sexual tissues (adrenal gland, bone marrow, brain, forestomach, heart, kidney, liver, large intestine, lung, muscle, small intestine, spleen, stomach, and thymus) and two sexual tissues (ovary and uterus for females or testis and vesicular gland for males) were excised. Each brain was cut in the sagittal plane giving rise to two samples per mouse. Two independent samples from liver, kidney and testis were also taken from each mouse. The excised samples were immediately soaked in RNA*later* solution (Thermo Fisher Scientific) for overnight. They were then transferred to 2.0 mL plastic tubes and stored at −80 °C. All the samples from the four mice are summarized in Supplementary Table S1. All the animal work was conducted according to the protocols and guidelines approved by the ethics committee of RIKEN Tsukuba Institute (Permit number: 11-014).

### Isolation of total RNA

Each tissue was ground (mortar and pestle, under continuous liquid N_2_ chilling) into a fine powder before RNA extraction. All the tissues were histologically confirmed to be normal, and stored at −80 °C. Total RNA was extracted from tissue by using the miRNeasy Mini Kit (Qiagen) according to the manufacturer’s protocol, including treatment with DNase. RNAs longer than 18 nucleotides were recovered with this method. RNA quality was evaluated with an Agilent Bioanalyzer (Agilent Technologies, Supplementary Table S1). All the samples had RNA integrity numbers (RINs) greater than 7, except for two bone marrow samples (RIN: 5.3 and 6.2) and one vesicular gland sample (RIN: 4.9). In addition, the RINs were not available of one small intestine sample and one large intestine sample. Excluding all the four bone marrow samples and the two samples of unknown RINs, the average RIN was 8.8 for the remaining 66 RNA samples.

### Construction and sequencing of RNA-seq libraries

We used a poly A selection protocol coupled with the Illumina TruSeq RNA-Seq library protocol to construct the mouse Bodymap RNA-Seq libraries. To monitor the quality of the transcriptomic profile data in the RNA-seq experiments, external RNA Control Consortium (ERCC) spike-in controls^45^ were added in each sample in an amount equivalent to about 1% of the mRNA content in the total RNA sample being sequenced before library construction. One single RNA-seq library was constructed for each RNA sample. Each library was sequenced using an Illumina HiScan platform (101 bp pair-end read). Eighteen different libraries (biological samples, randomized) were pooled together in equal amount and loaded onto two lanes of a flow cell (Supplementary Table S1), which would make two technical replicates from each biological sample. Reads from the two technical replicates of the same sample were combined together before we conducted the analyses.

### Read mapping and quantification

We first mapped the reads to the ERCC transcripts and the mouse Ensembl GRCm38 genome^46^ using Bowtie2^47^, allowing a maximum of two mismatches in the alignment. Then we used bedtools2^48^ and Cufflinks 2.2.1^49^ to quantify the ERCC transcripts and the mouse transcriptome, respectively. On average, 0.6% and 93.3% of the reads were mapped to the ERCC transcripts and the mouse genome, respectively (Supplementary Table S2). High correlations (R^2^ = 0.93) were found between the calculated ERCC levels and the theoretical values for both Mix 1 and Mix 2 (Supplementary Fig. S9) which indicated good reproducibility of RNA sequencing data. However, it was found that the correlations were strangely low between the two testis samples and any other samples. It turned out that reads mapped to ERCCs in the two testis samples were extremely low (4,686/0.02%, and 5,437/0.04% for each, respectively), indicating that the ERCCs might have not been added into these two samples. We also found that one of the bone marrow samples harbored abnormally high level of reads mapped to ERCCs (772,722/5.96%), implying that there might be RNA degradation with this sample (Supplementary Fig. S10).

### Analysis of transcriptomic gene-expression profiles

In our analyses, a gene or isoform was considered to be expressed in a sample if its FPKM was greater than 0.1 in the sample. In addition, a gene or isoform was considered ubiquitously expressed if its FPKM was greater than 0.1 across all samples. Genes with FPKM values of zero across all samples were removed, and the value of 1 was added to each FPKM value before log2 transformation to identify DEGs between different conditions. All the statistical analyses were conducted using R statistical programming language^50^.

### Identification of tissue-specific genes

Tissue-specific genes were identified using different FC thresholds of 2, 4, 8, 16, and 32 (Supplementary Table S4). In our analyses, a gene was considered to be a tissue-specific gene if its expression level was more than four-fold higher in a given tissue over any other tissues. Functional annotation was conducted using DAVID^24^ online tools. We also identified sex-dominated genes across all non-sexual tissues using a FC cutoff of 2.

### Identification of housekeeping genes

To identify housekeeping genes in mouse, we used the following criteria^32^: (i) highly expressed in all biological samples (FPKM > 1); (ii) low variance across tissues: standard-deviation [log2(FPKM)] <1; and (iii) no exceptional expression in any single tissue; that is, no logarithmic expression value differed from the averaged log2(FPKM) value by a factor of two (i.e. fourfold) or more. We also proposed a list of 27 genes as candidate reference genes using more rigorous criteria: (1) FPKM > 50 across all biological samples; (2) standard-deviation [log2(FPKM)] <0.5 over tissues; and (3) no expression value of any single tissue different from the averaged value across all tissues by twofold or more.

### Data availability

The datasets generated and analyzed during the current study are available in the NCBI SRA database repository (https://www.ncbi.nlm.nih.gov/bioproject/?term=PRJNA375882) and the Genome Sequence Archive (http://gsa.big.ac.cn/search/searchAll.action?term=PRJCA000427).

## Electronic supplementary material

Supplementary PDF File

Dataset 1
