# Supplementary material for: A Comprehensive Mouse Transcriptomic BodyMap across 17 Tissues by RNA-seq
Source: Sci Rep. 2017 Jun 23;7:4200. doi: 10.1038/s41598-017-04520-z (PMC5482823; doi:10.1038/s41598-017-04520-z)
Supplement: Supplementary file 1 — Supplementary PDF File [file 41598_2017_4520_MOESM1_ESM.pdf]

Supplementary Information

A Comprehensive Mouse Transcriptomic BodyMap across 17 Tissues by RNA-seq

Bin Li[1,2#], Tao Qing[1,2#], Jinhang Zhu[1], Zhuo Wen[1,3], Ying Yu[1,2], Ryutaro Fukumura[4], Yuanting Zheng[1,2*], Yoichi Gondo[4*], and Leming Shi[1,2*]

*To whom correspondence should be addressed (yuantingzheng@fudan.edu.cn, gondo@riken.jp, or lemingshi@fudan.edu.cn).

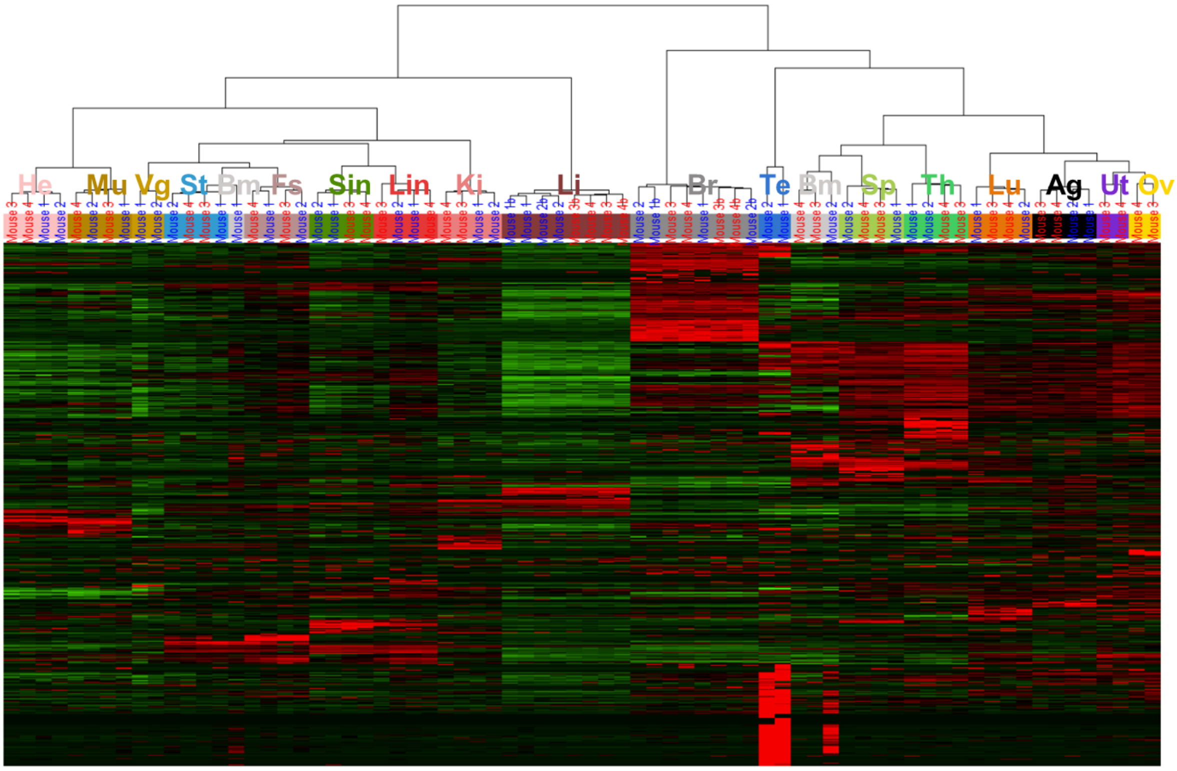

Supplementary Figure S1. Hierarchical clustering analysis of all 72 samples. Each column indicated a sample, whereas each row indicated a gene. Each tissue symbol was shown upon the color bar for each cluster. The mouse information where each sample came from was also labeled upon the color bar. Mouse 1 and Mouse 2 were two male mice (in blue text), while Mouse 3 and Mouse 4 were two female mice (in red text) as shown in Table 1. One of the bone marrow sample was not clustered together with the other three bone marrow samples, indicating that there may exist problems such as a mislabeling with this sample.

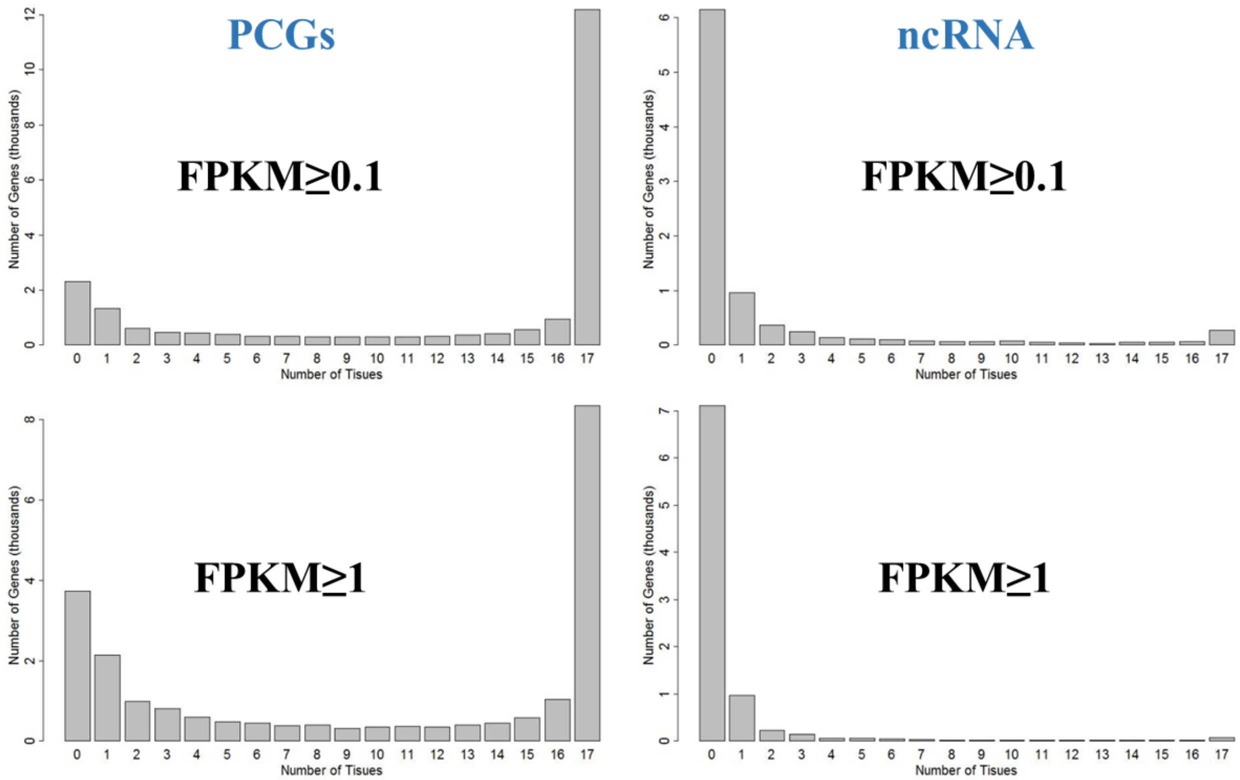

Supplementary Figure S2. Number of expressed genes under two different thresholds (0.1 and 1) given the number of tissues. FPKMs were averaged over all replicate samples in a given tissue. The gene expression distribution was complementary between PCGs (left panel) and ncRNA (right panel).

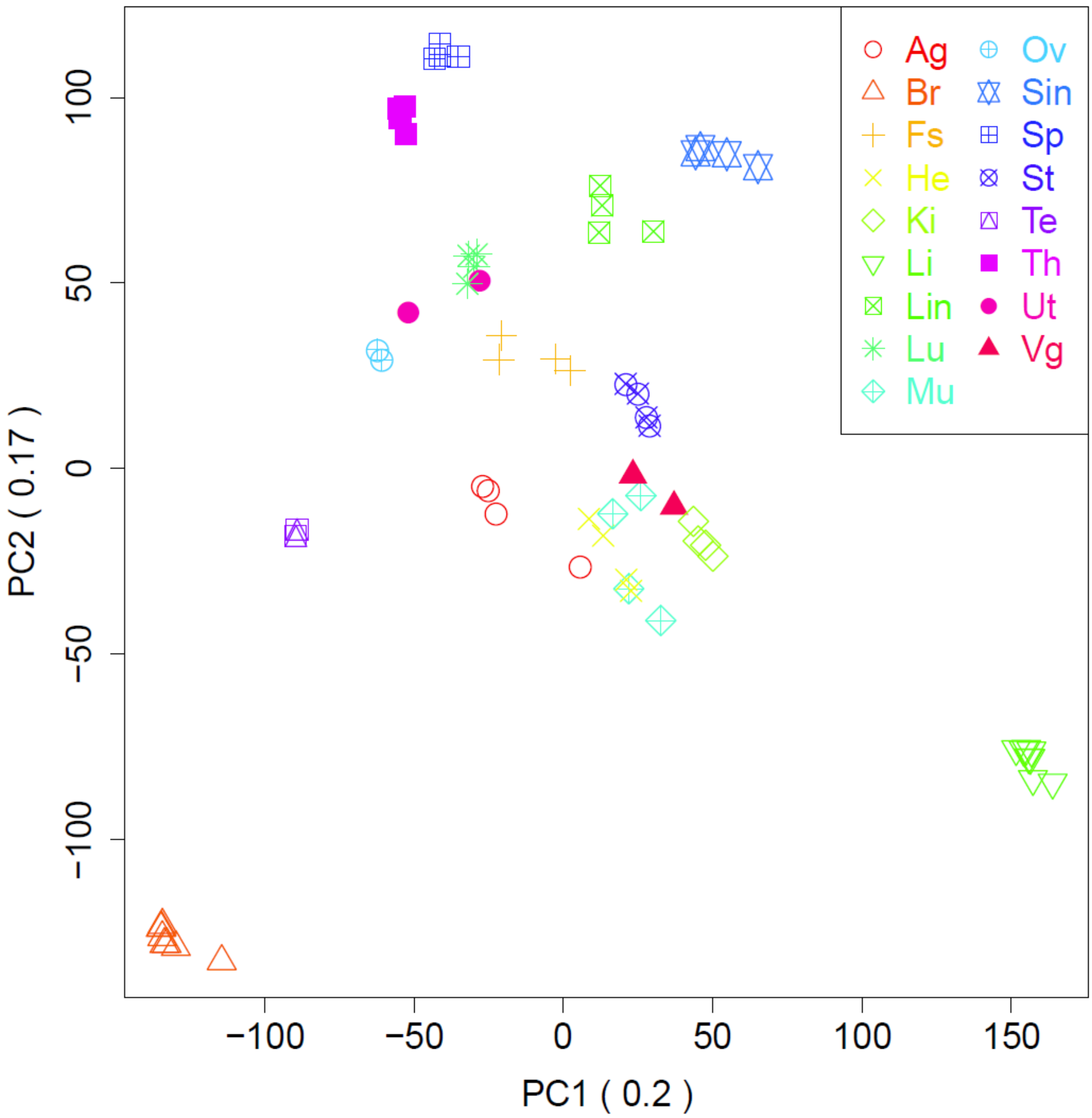

Supplementary Figure S3. Principal component analysis of the expression profiles of the 68 samples. Biological replicates from the same tissue were clustered together.

PCGs	He	Mu	Vg	St	Fs	Ki	Sin	Lin	Li	Br	Te	Sp	Th	Lu	Ag	Ut	Ov
He	0	674	585	2432	3716	2899	3604	3763	1763	5270	5496	5989	6543	5794	5103	1495	4642
Mu	657	0	513	2454	3887	2963	3619	3930	1698	5242	5640	5759	6111	5909	5169	1649	4936
Vg	821	872	0	831	1281	1321	1465	1328	944	2697	0	2516	2102	2462	1984	0	0
St	1531	1493	184	0	1027	2116	1821	1155	1294	4639	5125	4772	5433	4106	3877	862	3725
Fs	1024	1084	136	3	0	1588	1440	738	1111	3369	4303	2885	3775	2252	1833	124	1593
Ki	1217	1229	324	1476	2518	0	2201	2342	926	4111	4630	4340	5027	3940	2876	957	3091
Sin	2091	1996	413	1274	2904	2436	0	1079	1267	4834	5007	4391	5101	4407	4104	979	3674
Lin	1291	1261	175	516	1044	1737	821	0	1151	3874	4468	2967	4026	2826	2405	305	2101
Li	3269	3037	980	3697	6045	4271	4370	5607	0	6826	6254	7410	7533	7601	7421	1931	5470
Br	1794	1695	661	2268	2999	2728	3042	3135	1844	0	4144	3916	4395	3899	3124	1003	2404
Te	1786	1870	0	2150	3128	2416	2499	3028	1595	2967	0	3177	2775	3439	3161	0	0
Sp	1518	1572	443	1847	2442	2358	1981	2211	1433	3691	3820	0	1264	2638	2486	522	1542
Th	1967	1851	559	2185	2860	2730	2200	2599	1637	3739	3634	1664	0	3216	2922	636	1628
Lu	994	1173	247	1118	1310	1686	1802	1517	1186	3125	4074	1993	2800	0	1625	259	1054
Ag	890	981	329	1293	1664	1456	1935	1710	1065	3056	3774	2396	2999	2417	0	260	888
Ut	305	412	0	311	189	606	578	266	508	1123	0	633	473	433	264	0	0
Ov	470	636	0	527	593	792	915	657	646	1467	0	977	912	705	354	0	0

ncRNA	He	Mu	Vg	St	Fs	Ki	Sin	Lin	Li	Br	Te	Sp	Th	Lu	Ag	Ut	Ov
He	0	25	11	25	43	64	39	51	35	141	276	131	181	111	95	14	72
Mu	20	0	7	22	37	60	39	47	37	138	289	136	181	110	94	18	75
Vg	28	30	0	9	20	36	22	19	27	109	1	74	99	80	64	0	0
St	51	54	4	0	17	56	28	27	26	146	292	140	197	112	101	15	83
Fs	39	46	5	0	0	48	19	15	24	119	294	90	143	69	61	3	40
Ki	45	46	4	24	41	0	31	45	22	130	279	121	172	99	78	12	58
Sin	54	64	6	20	48	65	0	25	23	155	278	127	193	115	124	19	84
Lin	39	49	5	8	13	49	11	0	24	121	281	89	139	65	68	2	28
Li	78	71	19	46	75	82	54	94	0	193	304	187	227	167	164	37	109
Br	51	57	16	40	44	60	47	65	36	0	294	111	144	102	74	19	55
Te	32	44	0	25	37	46	29	46	27	97	0	87	95	77	66	0	0
Sp	41	53	9	26	31	56	23	40	26	115	259	0	56	71	63	5	29
Th	46	60	10	33	34	59	30	50	27	113	263	40	0	84	76	10	27
Lu	33	50	6	17	18	44	23	28	24	106	270	54	104	0	46	3	23
Ag	30	42	6	20	19	44	28	32	27	106	268	71	111	49	0	3	19
Ut	18	19	0	7	4	24	11	6	21	68	1	30	46	19	15	0	0
Ov	21	33	0	11	6	30	19	19	21	70	1	36	43	20	13	0	0

Supplementary Figure S4. Pairwise DEGs across all samples. Upper panel: protein-coding genes (PCGs); lower panel: ncRNA genes. For each tissue in each column, the figures indicated the numbers of down-regulated genes comparing this tissue to the other tissues. For each tissue in each row, the figures indicated the numbers of up-regulated genes comparing this tissue to the other tissues. The tissues are in the same order as in Figure 1C.

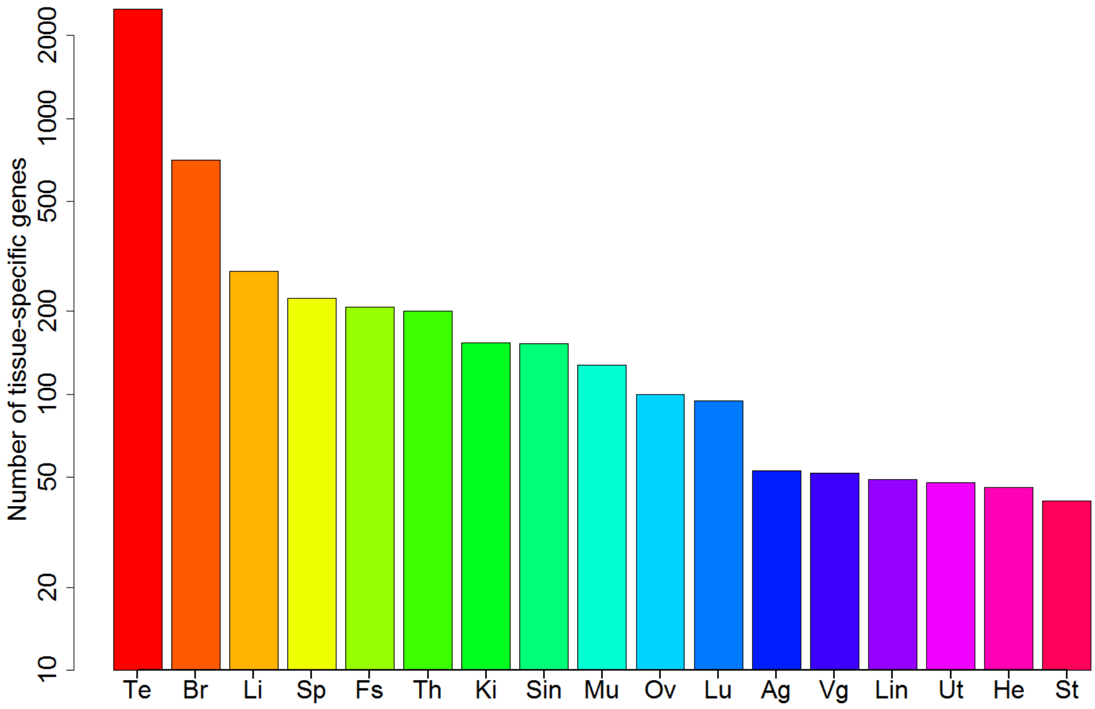

Supplementary Figure S5. Number of tissue-specific genes. Testis harbored the most tissue-specific genes (2,496), closely followed by brain (708) and liver (280). The numbers of tissue-specific genes identified in the other ten tissues ranged from 41 to 223.

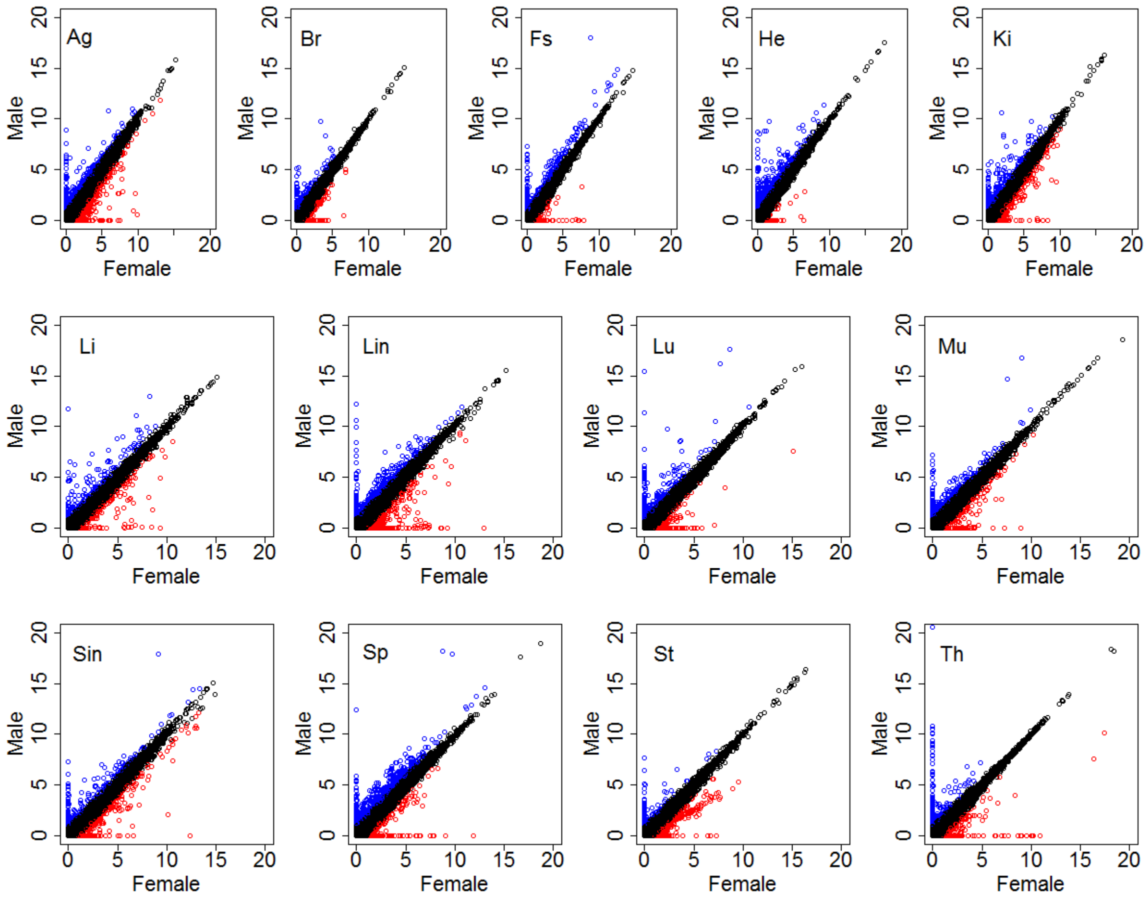

Supplementary Figure S6. Sex differences of rat transcriptomic profiles. Each scatter plot indicates the gene-expression level (expressed as log2FPKM) of male (y axis) against female (x axis) mouse for a given tissue among the thirteen non-sexual tissues. Male and female dominated genes are highlighted in blue and red, respectively.

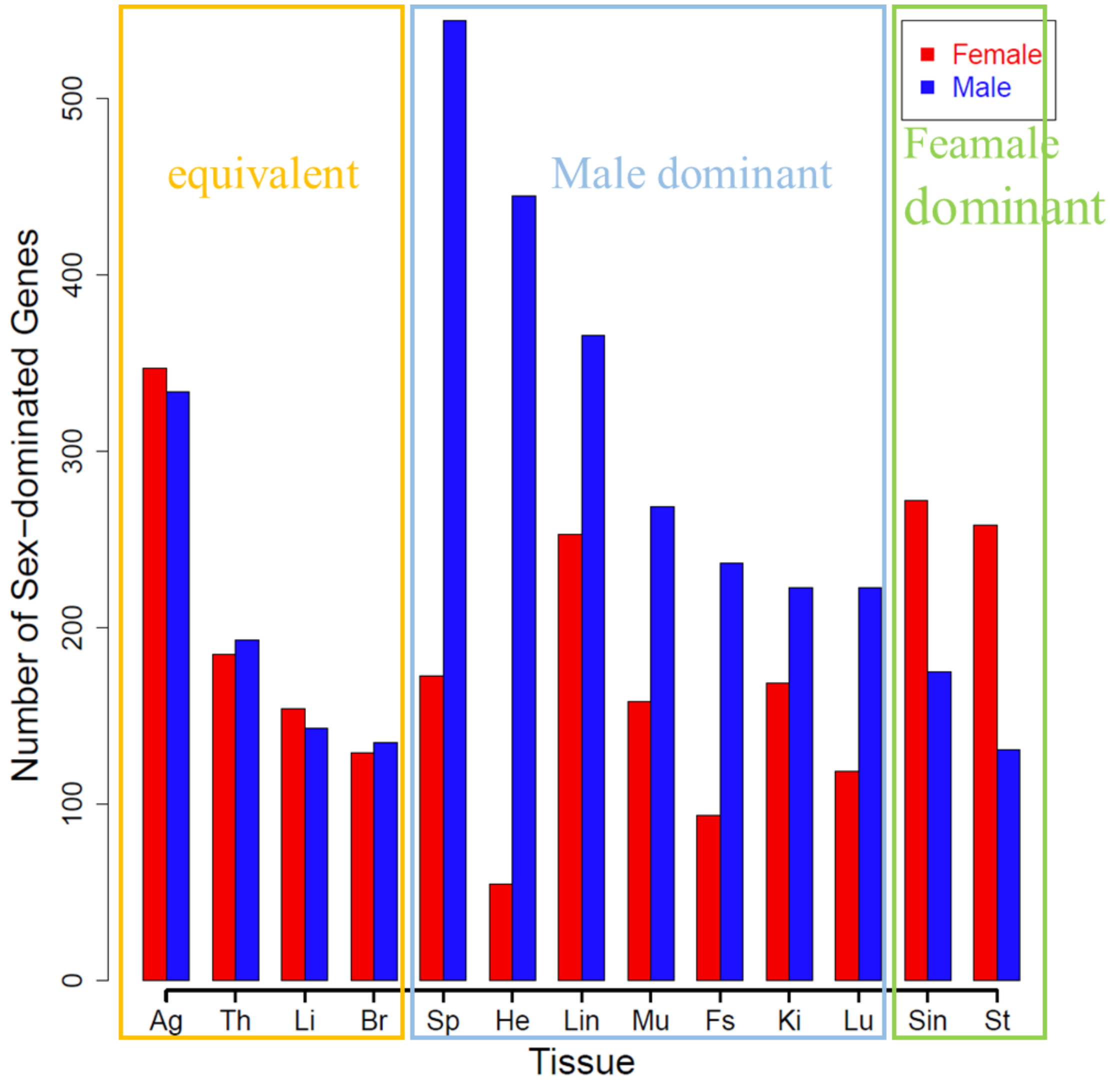

Supplementary Figure S7. Number of sex-dominated genes across all non-sexual tissues. Spleen, heart, large intestine, muscle, forestomach, kidney and lung expressed more male-dominated genes, whereas small intestine and stomach expressed more female-dominated genes.

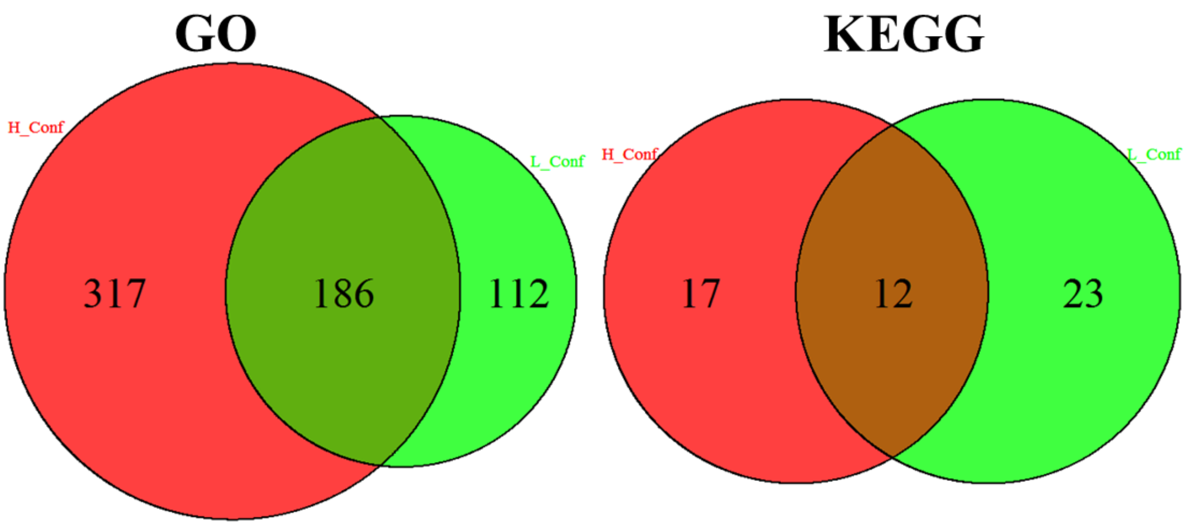

Supplementary Figure S8. Number of GO terms and KEGG pathways significantly enriched with the 4,781 house-keeping genes by groups. Red: number of GO terms or KEGG pathways by genes with high confidence; Green: number of GO terms or KEGG pathways genes with relatively low confidence. Left panel: GO terms, right panel: KEGG pathways.

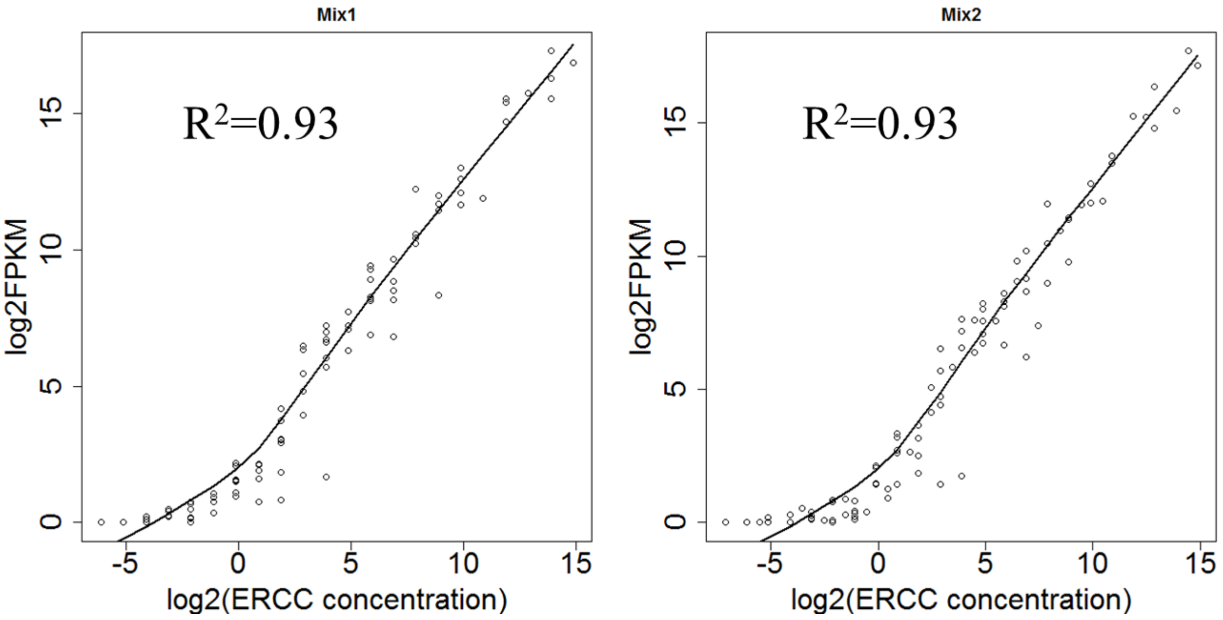

Supplementary Figure S9. High correlation found between the calculated ERCC levels (y axis, log2FPKM) and the theoretical values (x axis, log2[ERCC concentration]). Left panel: Mix 1, right panel: Mix 2. The calculated values agreed well with the theoretical ones (R^2=0.93).

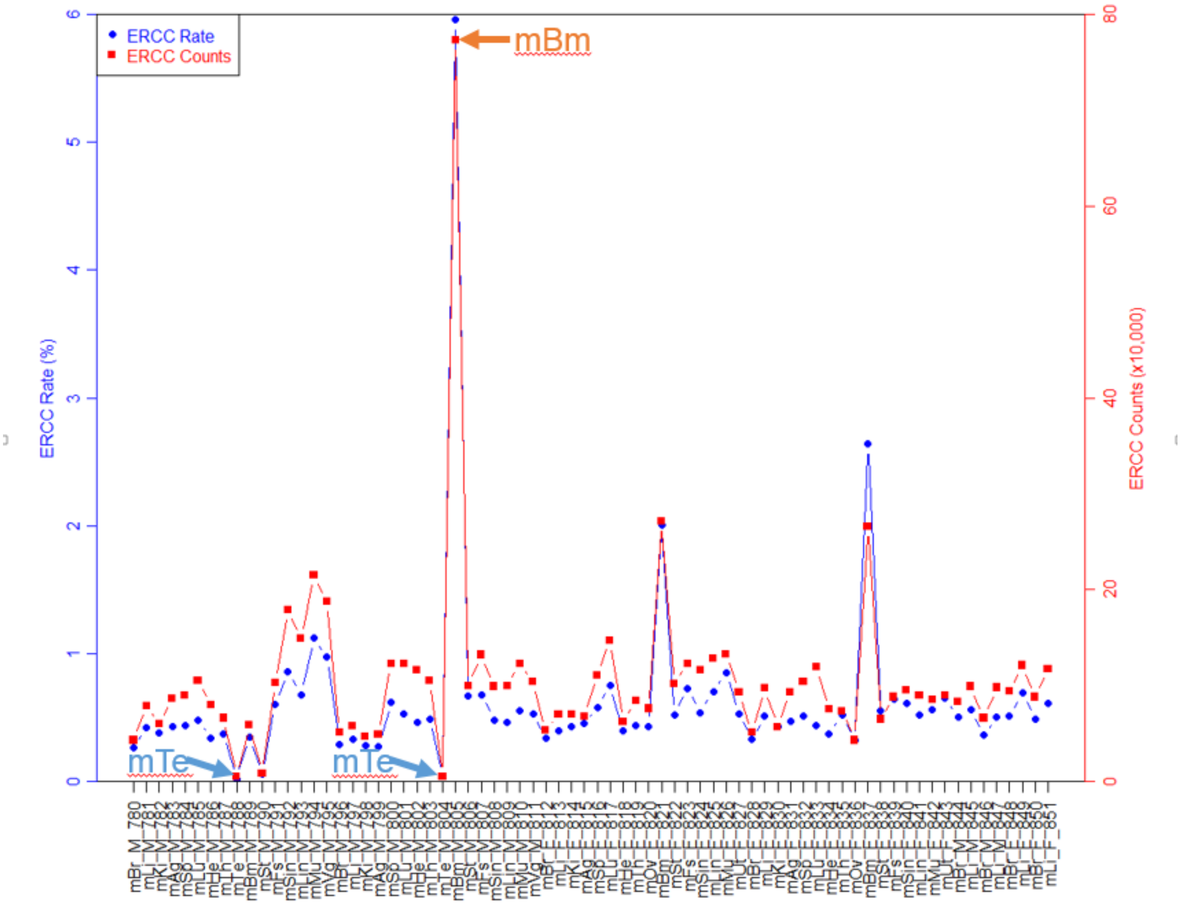

Supplementary Figure S10. ERCC mapping ration and counts. Left y axis: ERCC rate (the blue line); Right y axis: ERCC counts (the red line).

Supplementary Table S1. Isolation of total RNA.

Order_RNA	ProcessingOrder_RNA_Seq	UniqueSampeID	Con(ng/ul)	260/280	Yield (ug)	RIN	For_RNA-Seq	ERCC_Mix	Pool
**1**	1	mBr_M_6w_1	712.6	2.12	35.6	8.2	YES	Mix1	Pool_1
**3**	2	mLi_M_6w_1	2274.1	2.06	113.7	8.6	YES	Mix1	Pool_1
**5**	3	mKi_M_6w_1	1250.5	2.11	62.5	8.1	YES	Mix1	Pool_1
**7**	4	mAg_M_6w_1	104.6	2.14	5.2	8.3	YES	Mix1	Pool_1
**8**	5	mSp_M_6w_1	784.4	2.11	39.2	9.4	YES	Mix1	Pool_1
**9**	6	mLu_M_6w_1	123.3	2.09	6.2	8.4	YES	Mix1	Pool_1
**10**	7	mHe_M_6w_1	523.3	2.14	26.2	7.9	YES	Mix1	Pool_1
**11**	8	mTh_M_6w_1	1081.1	2.12	54.1	9.8	YES	Mix1	Pool_1
**12**	9	mTe_M_6w_1	711.0	2.14	35.5	9.1	YES	Mix1	Pool_1
**14**	10	mBm_M_6w_1	447.7	2.05	22.4	7.2	YES	Mix1	Pool_1
**15**	11	mSt_M_6w_1	876.2	2.1	43.8	8.6	YES	Mix1	Pool_1
**16**	12	mFs_M_6w_1	428.5	2.06	21.4	8.9	YES	Mix1	Pool_1
**17**	13	mSin_M_6w_1	2077.3	2.08	103.9	8.5	YES	Mix2	Pool_1
**18**	14	mLin_M_6w_1	477.2	2.04	23.9	9.2	YES	Mix2	Pool_1
**19**	15	mMu_M_6w_1	70.6	2.14	3.5	7.5	YES	Mix2	Pool_1
**20**	16	mVg_M_6w_1	998.3	2.09	49.9	4.9	YES	Mix2	Pool_1
**21**	17	mBr_M_6w_2	772.6	2.14	38.6	8.7	YES	Mix2	Pool_1
**23**	18	mLi_M_6w_2	2032.6	2.08	101.6	9.1	YES	Mix2	Pool_1
**25**	19	mKi_M_6w_2	693.7	2.16	34.7	8.2	YES	Mix2	Pool_2
**27**	20	mAg_M_6w_2	127.7	2.15	6.4	9	YES	Mix2	Pool_2
**28**	21	mSp_M_6w_2	889.1	2.11	44.5	9.6	YES	Mix2	Pool_2
**29**	22	mLu_M_6w_2	159.2	2.13	8.0	8.5	YES	Mix2	Pool_2
**30**	23	mHe_M_6w_2	176.6	2.14	8.8	8.9	YES	Mix2	Pool_2
**31**	24	mTh_M_6w_2	768.0	2.12	38.4	9.9	YES	Mix2	Pool_2
**32**	25	mTe_M_6w_2	814.0	2.1	40.7	9.5	YES	Mix1	Pool_2
**34**	26	mBm_M_6w_2	426.8	2.06	21.3	5.3	YES	Mix1	Pool_2
**35**	27	mSt_M_6w_2	664.4	2.13	33.2	8.7	YES	Mix1	Pool_2
**36**	28	mFs_M_6w_2	262.2	2.09	13.1	9.1	YES	Mix1	Pool_2
**37**	29	mSin_M_6w_2	1613.1	2.1	80.7	9.7	YES	Mix1	Pool_2
**38**	30	mLin_M_6w_2	269.3	2.11	13.5	9.3	YES	Mix1	Pool_2
**39**	31	mMu_M_6w_2	308.3	2.1	15.4	8.7	YES	Mix1	Pool_2
**40**	32	mVg_M_6w_2	638.1	2.1	31.9	7	YES	Mix1	Pool_2
**41**	33	mBr_F_6w_3	392.3	2.07	19.6	8.7	YES	Mix1	Pool_2
**43**	34	mLi_F_6w_3	2273.8	2.08	113.7	8.9	YES	Mix1	Pool_2
**45**	35	mKi_F_6w_3	652.8	2.14	32.6	8.6	YES	Mix1	Pool_2
**47**	36	mAg_F_6w_3	183.2	2.1	9.2	9.4	YES	Mix1	Pool_2
**48**	37	mSp_F_6w_3	718.0	2.13	35.9	9.2	YES	Mix2	Pool_3
**49**	38	mLu_F_6w_3	102.7	1.85	5.1	8.9	YES	Mix2	Pool_3
**50**	39	mHe_F_6w_3	121.7	2.1	6.1	9.2	YES	Mix2	Pool_3
**51**	40	mTh_F_6w_3	743.0	2.13	37.1	9.9	YES	Mix2	Pool_3

**52**	41	mOv_F_6w_3	296.7	2.09	14.8	9.8	YES	Mix2	Pool_3
**53**	42	mBm_F_6w_3	528.9	2.14	26.4	7.2	YES	Mix2	Pool_3
**54**	43	mSt_F_6w_3	319.6	2.08	16.0	9.1	YES	Mix2	Pool_3
**55**	44	mFs_F_6w_3	146.4	2.09	7.3	9.5	YES	Mix2	Pool_3
**56**	45	mSin_F_6w_3	771.9	2.14	38.6	NA	YES	Mix2	Pool_3
**57**	46	mLin_F_6w_3	597.7	2.15	29.9	NA	YES	Mix2	Pool_3
**58**	47	mMu_F_6w_3	43.2	2.04	2.2	8.2	YES	Mix2	Pool_3
**59**	48	mUt_F_6w_3	456.6	2.04	22.8	10	YES	Mix2	Pool_3
**60**	49	mBr_F_6w_4	525.7	2.17	26.3	8.5	YES	Mix1	Pool_3
**62**	50	mLi_F_6w_4	2161.4	2.08	108.1	8.7	YES	Mix1	Pool_3
**64**	51	mKi_F_6w_4	779.9	2.15	39.0	8.5	YES	Mix1	Pool_3
**66**	52	mAg_F_6w_4	201.2	2.11	10.1	9.2	YES	Mix1	Pool_3
**67**	53	mSp_F_6w_4	466.4	2.04	23.3	9.6	YES	Mix1	Pool_3
**68**	54	mLu_F_6w_4	106.2	2.1	5.3	8.7	YES	Mix1	Pool_3
**69**	55	mHe_F_6w_4	155.6	2.1	7.8	8.7	YES	Mix1	Pool_4
**70**	56	mTh_F_6w_4	862.7	2.15	43.1	10	YES	Mix1	Pool_4
**71**	57	mOv_F_6w_4	326.9	2.09	16.3	9.9	YES	Mix1	Pool_4
**72**	58	mBm_F_6w_4	559.8	2.16	28.0	6.2	YES	Mix1	Pool_4
**73**	59	mSt_F_6w_4	1259.6	2.12	63.0	9.1	YES	Mix1	Pool_4
**74**	60	mFs_F_6w_4	290.5	2.08	14.5	9.2	YES	Mix1	Pool_4
**75**	61	mSin_F_6w_4	681.8	2.15	34.1	8.8	YES	Mix2	Pool_4
**76**	62	mLin_F_6w_4	474.5	2.04	23.7	9.4	YES	Mix2	Pool_4
**77**	63	mMu_F_6w_4	192.3	2.1	9.6	8.6	YES	Mix2	Pool_4
**78**	64	mUt_F_6w_4	220.0	2.08	11.0	9.1	YES	Mix2	Pool_4
**2**	65	mBr_M_6w_1b	1046.6	2.12	52.3	7.9	YES	Mix2	Pool_4
**4**	66	mLi_M_6w_1b	1637.3	2.1	81.9	8.2	YES	Mix2	Pool_4
**22**	67	mBr_M_6w_2b	1000.3	2.12	50.0	8.8	YES	Mix2	Pool_4
**24**	68	mLi_M_6w_2b	1450.9	2.1	72.5	8.9	YES	Mix2	Pool_4
**42**	69	mBr_F_6w_3b	656.3	2.14	32.8	8.8	YES	Mix2	Pool_4
**44**	70	mLi_F_6w_3b	1662.4	2.1	83.1	8.9	YES	Mix2	Pool_4
**61**	71	mBr_F_6w_4b	550.3	2.16	27.5	8.6	YES	Mix2	Pool_4
**63**	72	mLi_F_6w_4b	1677.2	2.11	83.9	8.9	YES	Mix2	Pool_4
**6**	Ignore	mKi_M_6w_1b	1008.46	2.11	50.423	7.9	NO		
**13**	Ignore	mTe_M_6w_1b	820.83	2.14	41.0415	8.5	NO		
**26**	Ignore	mKi_M_6w_2b	427.54	2.09	21.377	8.6	NO		
**33**	Ignore	mTe_M_6w_2b	622.5	2.09	31.125	9.3	NO		
**46**	Ignore	mKi_F_6w_3b	669.15	2.12	33.4575	8.7	NO		
**65**	Ignore	mKi_F_6w_4b	406.41	2.07	20.3205	8.9	NO		

The "UniqueSampleID" column was used for naming raw fastq data files. For each sample ID, for example, "mBr_M_6w_1", "mBr" stands for tissue (including 18 different types of tissues), "M" stands for sex (M for male and F for female), "6w" stands for age, "1" stands

for animal replicates (including two male and two female mice, numbered as 1,2,3,4), and “b” stands for RNA sample replicate which only occurred in brain, liver, kidney, and testis. It should be noted that each pool was loaded onto two lanes of a flow cell for sequencing.

Supplementary Table S2. Read counts summary and mapping stats of all samples.

SampleID	ReadCounts	ReadsAfterTrimming	ReadsRemoved	TrimmingRate (%)	OverallMappingRate (%)	ERCCReads	ERCCRate (%)
**mBr_M_6w_1**	18525008	16666932	1858076	10.0	94.5	42709	0.26
**mLi_M_6w_1**	20896688	19027264	1869424	8.9	94.1	79268	0.42
**mKi_M_6w_1**	17537336	15892748	1644588	9.4	89.9	60483	0.38
**mAg_M_6w_1**	22064332	19877900	2186432	9.9	93.2	85862	0.43
**mSp_M_6w_1**	22572530	20299026	2273504	10.1	96.0	89832	0.44
**mLu_M_6w_1**	24525136	22171380	2353756	9.6	95.0	105411	0.48
**mHe_M_6w_1**	25844398	23539150	2305248	8.9	84.3	80297	0.34
**mTh_M_6w_1**	19361654	17509262	1852392	9.6	96.0	65509	0.37
**mTe_M_6w_1**	21161906	19059702	2102204	9.9	96.4	4686	0.02
**mBm_M_6w_1***	18575368	16732044	1843324	9.9	93.9	59114	0.35
**mSt_M_6w_1**	15190592	13692732	1497860	9.9	93.7	8258	0.06
**mFs_M_6w_1**	19038200	17144340	1893860	9.9	94.1	102698	0.60
**mSin_M_6w_1**	23035516	20816606	2218910	9.6	93.2	178921	0.86
**mLin_M_6w_1**	24050944	21725582	2325362	9.7	93.9	148762	0.68
**mMu_M_6w_1**	21158214	19114222	2043992	9.7	88.7	214882	1.12
**mVg_M_6w_1**	21091328	19307924	1783404	8.5	95.5	188221	0.97
**mBr_M_6w_2**	19571314	17606970	1964344	10.0	93.2	50891	0.29
**mLi_M_6w_2**	19258190	17498838	1759352	9.1	94.7	57815	0.33
**mKi_M_6w_2**	18584284	16727752	1856532	10.0	89.2	47271	0.28
**mAg_M_6w_2**	20322612	18152340	2170272	10.7	93.8	49202	0.27
**mSp_M_6w_2**	22575994	20071408	2504586	11.1	95.5	123448	0.62
**mLu_M_6w_2**	26140480	23398842	2741638	10.5	94.8	123289	0.53
**mHe_M_6w_2**	27726914	24968424	2758490	9.9	85.5	115635	0.46
**mTh_M_6w_2**	24032546	21531988	2500558	10.4	95.8	105293	0.49
**mTe_M_6w_2**	17177416	15367784	1809632	10.5	96.6	5437	0.04
**mBm_M_6w_2***	14727854	12954378	1773476	12.0	87.4	772722	5.96
**mSt_M_6w_2**	16939036	14983438	1955598	11.5	93.2	100153	0.67

**mFs_M_6w_2**	21871886	19367078	2504808	11.5	94.7	132166	0.68
**mSin_M_6w_2**	23133664	20731946	2401718	10.4	93.8	98747	0.48
**mLin_M_6w_2**	24336470	21756286	2580184	10.6	94.3	99534	0.46
**mMu_M_6w_2**	24912298	22269556	2642742	10.6	91.0	122642	0.55
**mVg_M_6w_2**	21489520	19497926	1991594	9.3	95.3	103866	0.53
**mBr_F_6w_3**	17254978	15334488	1920490	11.1	93.8	52725	0.34
**mLi_F_6w_3**	19512402	17621396	1891006	9.7	93.8	70065	0.40
**mKi_F_6w_3**	17806624	16036380	1770244	9.9	89.4	69646	0.43
**mAg_F_6w_3**	17500164	14982954	2517210	14.4	93.0	67665	0.45
**mSp_F_6w_3**	21162944	19018126	2144818	10.1	95.7	110531	0.58
**mLu_F_6w_3**	21681456	19519366	2162090	10.0	94.8	147120	0.75
**mHe_F_6w_3**	17178324	15488782	1689542	9.8	82.9	61860	0.40
**mTh_F_6w_3**	21241628	19089936	2151692	10.1	96.2	84076	0.44
**mOv_F_6w_3**	19771088	17762660	2008428	10.2	95.7	75502	0.43
**mBm_F_6w_3***	15088784	13471096	1617688	10.7	93.9	270805	2.01
**mSt_F_6w_3**	21704066	19460376	2243690	10.3	93.2	101977	0.52
**mFs_F_6w_3**	18922354	16884352	2038002	10.8	95.2	122625	0.73
**mSin_F_6w_3**	23701666	21340548	2361118	10.0	94.6	115757	0.54
**mLin_F_6w_3**	20223568	18334958	1888610	9.3	94.2	127844	0.70
**mMu_F_6w_3**	17229904	15615182	1614722	9.4	90.0	132699	0.85
**mUt_F_6w_3**	19656324	17676074	1980250	10.1	95.6	93205	0.53
**mBr_F_6w_4**	17364056	15640986	1723070	9.9	93.9	51288	0.33
**mLi_F_6w_4**	20961492	19033962	1927530	9.2	93.9	97358	0.51
**mKi_F_6w_4**	14610436	13228868	1381568	9.5	89.2	57355	0.43
**mAg_F_6w_4**	22178632	19875496	2303136	10.4	93.4	92875	0.47
**mSp_F_6w_4**	22704312	20429870	2274442	10.0	95.5	104431	0.51
**mLu_F_6w_4**	30394904	27361452	3033452	10.0	95.5	119353	0.44
**mHe_F_6w_4**	22442576	19967770	2474806	11.0	84.7	74741	0.37
**mTh_F_6w_4**	15835926	13960316	1875610	11.8	95.9	72853	0.52

**mOv_F_6w_4**	15652612	13737262	1915350	12.2	95.8	43311	0.32
**mBm_F_6w_4***	11501782	10044622	1457160	12.7	93.4	265612	2.64
**mSt_F_6w_4**	13450798	11817084	1633714	12.1	93.1	64718	0.55
**mFs_F_6w_4**	15771670	13805992	1965678	12.5	94.1	87938	0.64
**mSin_F_6w_4**	17692576	15585984	2106592	11.9	94.2	95132	0.61
**mLin_F_6w_4**	19658454	17304712	2353742	12.0	94.0	89988	0.52
**mMu_F_6w_4**	17423318	15277286	2146032	12.3	89.6	84891	0.56
**mUt_F_6w_4**	15952878	13972728	1980150	12.4	95.3	90466	0.65
**mBr_M_6w_1b**	18964948	16635036	2329912	12.3	93.3	83324	0.50
**mLi_M_6w_1b**	19852936	17688740	2164196	10.9	94.0	99311	0.56
**mBr_M_6w_2b**	21200474	18601370	2599104	12.3	94.3	66088	0.36
**mLi_M_6w_2b**	22397662	19716346	2681316	12.0	94.7	98445	0.50
**mBr_F_6w_3b**	20889464	18358194	2531270	12.1	93.3	94463	0.51
**mLi_F_6w_3b**	19807288	17628090	2179198	11.0	93.8	121069	0.69
**mBr_F_6w_4b**	20275116	17863378	2411738	11.9	93.6	87798	0.49
**mLi_F_6w_4b**	21432152	19046116	2386036	11.1	93.8	116927	0.61

*The four bone marrow samples were removed when the analyses were conducted.

Supplementary Table S3. Significantly (P value < 0.05) enriched GO terms based on tissue-specific genes across all 17 tissues. Only top 10 terms of each tissue were listed. BP: biological process, CC: cellular component, MF: molecular function.

Tissue	Category	Term	Count	PValue
**Ag**	BP	GO:0006694~steroid biosynthetic process	8	3.01E-10
**Ag**	BP	GO:0008202~steroid metabolic process	9	3.52E-09
**Ag**	BP	GO:0055114~oxidation reduction	13	2.64E-08
**Ag**	BP	GO:0007631~feeding behavior	6	0.000000134
**Ag**	BP	GO:0008610~lipid biosynthetic process	9	0.000000299
**Ag**	BP	GO:0034754~cellular hormone metabolic process	5	0.00000719
**Ag**	BP	GO:0042446~hormone biosynthetic process	4	0.0000168
**Ag**	BP	GO:0007610~behavior	8	0.0000433
**Ag**	BP	GO:0042756~drinking behavior	3	0.0000569
**Ag**	BP	GO:0042445~hormone metabolic process	5	0.0000636
**Br**	CC	GO:0045202~synapse	100	6.66E-62
**Br**	CC	GO:0044456~synapse part	77	1.62E-52
**Br**	CC	GO:0043005~neuron projection	77	3.14E-47
**Br**	BP	GO:0019226~transmission of nerve impulse	65	1.35E-43
**Br**	BP	GO:0007268~synaptic transmission	55	1.49E-38
**Br**	CC	GO:0005886~plasma membrane	237	7.61E-33
**Br**	BP	GO:0007267~cell-cell signaling	61	1.82E-32
**Br**	CC	GO:0030054~cell junction	82	4.8E-30
**Br**	BP	GO:0006836~neurotransmitter transport	33	1.47E-27
**Br**	CC	GO:0045211~postsynaptic membrane	42	1.22E-26
**Fs**	BP	GO:0030216~keratinocyte differentiation	15	4.32E-20
**Fs**	BP	GO:0031424~keratinization	13	9.23E-20
**Fs**	BP	GO:0009913~epidermal cell differentiation	15	1.14E-19
**Fs**	BP	GO:0008544~epidermis development	19	1.33E-19
**Fs**	BP	GO:0007398~ectoderm development	19	4.23E-19

**Fs**	BP	GO:0030855~epithelial cell differentiation	17	8.83E-17
**Fs**	CC	GO:0001533~cornified envelope	10	7.25E-15
**Fs**	CC	GO:0030057~desmosome	9	1.35E-12
**Fs**	BP	GO:0060429~epithelium development	17	2.31E-11
**Fs**	MF	GO:0030414~peptidase inhibitor activity	15	3.77E-11
**He**	BP	GO:0007507~heart development	11	4.89E-12
**He**	CC	GO:0030016~myofibril	9	8.07E-12
**He**	CC	GO:0043292~contractile fiber	9	1.15E-11
**He**	CC	GO:0030017~sarcomere	7	1.18E-08
**He**	CC	GO:0044449~contractile fiber part	7	1.83E-08
**He**	BP	GO:0008016~regulation of heart contraction	6	3.41E-08
**He**	BP	GO:0048738~cardiac muscle tissue development	6	6.68E-08
**He**	BP	GO:0044057~regulation of system process	8	7.24E-08
**He**	BP	GO:0002026~regulation of the force of heart contraction	4	0.00000176
**He**	BP	GO:0008015~blood circulation	6	0.00000191
**Ki**	BP	GO:0006811~ion transport	33	2.95E-19
**Ki**	BP	GO:0055085~transmembrane transport	26	7.04E-17
**Ki**	MF	GO:0008509~anion transmembrane transporter activity	17	1.07E-15
**Ki**	BP	GO:0006820~anion transport	16	3.93E-15
**Ki**	CC	GO:0045177~apical part of cell	17	6.96E-15
**Ki**	CC	GO:0016324~apical plasma membrane	15	2.79E-14
**Ki**	MF	GO:0015293~symporter activity	14	1.16E-11
**Ki**	BP	GO:0006812~cation transport	21	7.26E-11
**Ki**	MF	GO:0031402~sodium ion binding	12	2.01E-10
**Ki**	BP	GO:0006814~sodium ion transport	12	4.5E-10
**Li**	CC	GO:0005576~extracellular region	94	8.53E-35
**Li**	BP	GO:0009611~response to wounding	41	1.8E-26
**Li**	MF	GO:0020037~heme binding	30	2.88E-25
**Li**	MF	GO:0046906~tetrapyrrole binding	30	1.21E-24

**Li**	CC	GO:0005615~extracellular space	47	2.51E-24
**Li**	MF	GO:0016712~oxidoreductase activity	20	4.93E-24
**Li**	BP	GO:0002526~acute inflammatory response	23	2.74E-23
**Li**	MF	GO:0009055~electron carrier activity	30	6.34E-21
**Li**	MF	GO:0004866~endopeptidase inhibitor activity	27	3.61E-20
**Li**	MF	GO:0005506~iron ion binding	36	3.84E-20
**Lin**	MF	GO:0005179~hormone activity	4	0.000712
**Lin**	MF	GO:0008509~anion transmembrane transporter activity	4	0.00107
**Lin**	BP	GO:0015698~inorganic anion transport	3	0.00472
**Lin**	MF	GO:0031404~chloride ion binding	3	0.00509
**Lin**	MF	GO:0043168~anion binding	3	0.00538
**Lin**	BP	GO:0070085~glycosylation	3	0.00565
**Lin**	BP	GO:0043413~biopolymer glycosylation	3	0.00565
**Lin**	BP	GO:0006486~protein amino acid glycosylation	3	0.00565
**Lin**	BP	GO:0009101~glycoprotein biosynthetic process	3	0.00919
**Lin**	CC	GO:0005576~extracellular region	9	0.0105
**Lu**	MF	GO:0030246~carbohydrate binding	10	0.00000093
**Lu**	MF	GO:0005529~sugar binding	7	0.0000308
**Lu**	CC	GO:0005576~extracellular region	21	0.0000643
**Lu**	BP	GO:0007585~respiratory gaseous exchange	4	0.0000742
**Lu**	BP	GO:0030324~lung development	5	0.000486
**Lu**	BP	GO:0030323~respiratory tube development	5	0.000519
**Lu**	BP	GO:0006022~aminoglycan metabolic process	4	0.00067
**Lu**	CC	GO:0005615~extracellular space	10	0.000682
**Lu**	BP	GO:0060541~respiratory system development	5	0.000737
**Lu**	CC	GO:0044421~extracellular region part	12	0.001
**Mu**	CC	GO:0030017~sarcomere	14	1.08E-16
**Mu**	CC	GO:0044449~contractile fiber part	14	2.91E-16
**Mu**	CC	GO:0030016~myofibril	14	6.27E-16

**Mu**	CC	GO:0043292~contractile fiber	14	1.12E-15
**Mu**	CC	GO:0016529~sarcoplasmic reticulum	9	4.9E-12
**Mu**	CC	GO:0016528~sarcoplasm	9	8.1E-12
**Mu**	BP	GO:0006006~glucose metabolic process	12	8.68E-11
**Mu**	BP	GO:0005977~glycogen metabolic process	8	2.21E-10
**Mu**	BP	GO:0044042~glucan metabolic process	8	2.21E-10
**Mu**	BP	GO:0006073~cellular glucan metabolic process	8	2.21E-10
**Ov**	BP	GO:0019953~sexual reproduction	10	0.00000213
**Ov**	BP	GO:0048608~reproductive structure development	7	0.0000027
**Ov**	BP	GO:0009566~fertilization	6	0.00000284
**Ov**	BP	GO:0008406~gonad development	6	0.00000715
**Ov**	BP	GO:0045137~development of primary sexual characteristics	6	0.0000162
**Ov**	BP	GO:0008585~female gonad development	5	0.0000207
**Ov**	CC	GO:0005576~extracellular region	17	0.0000272
**Ov**	BP	GO:0046545~development of primary female sexual characteristics	5	0.0000277
**Ov**	BP	GO:0046660~female sex differentiation	5	0.0000438
**Ov**	BP	GO:0007548~sex differentiation	6	0.0000475
**Sin**	BP	GO:0006952~defense response	16	6.4E-09
**Sin**	CC	GO:0005903~brush border	7	4.56E-08
**Sin**	BP	GO:0042742~defense response to bacterium	8	0.00000131
**Sin**	BP	GO:0009617~response to bacterium	9	0.00000137
**Sin**	MF	GO:0008238~exopeptidase activity	7	0.00000222
**Sin**	MF	GO:0004177~aminopeptidase activity	5	0.0000155
**Sin**	CC	GO:0005576~extracellular region	24	0.0000514
**Sin**	MF	GO:0008237~metallopeptidase activity	8	0.0000727
**Sin**	CC	GO:0005615~extracellular space	11	0.00073
**Sin**	CC	GO:0042612~MHC class I protein complex	4	0.00186
**Sp**	BP	GO:0006955~immune response	34	2.15E-22
**Sp**	BP	GO:0009611~response to wounding	22	4.64E-13

**Sp**	BP	GO:0050778~positive regulation of immune response	15	4.17E-12
**Sp**	BP	GO:0006952~defense response	23	7.84E-12
**Sp**	BP	GO:0002253~activation of immune response	12	8.67E-11
**Sp**	BP	GO:0002684~positive regulation of immune system process	16	1.02E-10
**Sp**	BP	GO:0001775~cell activation	17	1.26E-10
**Sp**	BP	GO:0048584~positive regulation of response to stimulus	15	2.9E-10
**Sp**	BP	GO:0006954~inflammatory response	15	3.51E-09
**Sp**	BP	GO:0002252~immune effector process	12	5.5E-09
**St**	BP	GO:0006885~regulation of pH	3	0.00113
**St**	MF	GO:0004190~aspartic-type endopeptidase activity	3	0.00116
**St**	MF	GO:0070001~aspartic-type peptidase activity	3	0.00116
**St**	CC	GO:0005576~extracellular region	12	0.00117
**St**	BP	GO:0055067~monovalent inorganic cation homeostasis	3	0.0017
**St**	BP	GO:0055080~cation homeostasis	4	0.00311
**St**	BP	GO:0010155~regulation of proton transport	2	0.00324
**St**	BP	GO:0006811~ion transport	6	0.00487
**St**	BP	GO:0030001~metal ion transport	5	0.00507
**St**	MF	GO:0008900~hydrogen:potassium-exchanging ATPase activity	2	0.00563
**Te**	BP	GO:0019953~sexual reproduction	121	1.63E-70
**Te**	BP	GO:0048232~male gamete generation	100	1.76E-68
**Te**	BP	GO:0007283~spermatogenesis	100	1.76E-68
**Te**	BP	GO:0007276~gamete generation	103	3.42E-59
**Te**	BP	GO:0048609~reproductive process in a multicellular organism	107	2.7E-53
**Te**	BP	GO:0032504~multicellular organism reproduction	107	2.7E-53
**Te**	CC	GO:0019861~flagellum	32	3.64E-28
**Te**	CC	GO:0001669~acrosomal vesicle	27	6.38E-25
**Te**	BP	GO:0048610~reproductive cellular process	46	6.74E-23
**Te**	CC	GO:0005929~cilium	39	7.66E-21
**Th**	BP	GO:0030217~T cell differentiation	14	1.47E-17

**Th**	BP	GO:0042110~T cell activation	15	1.25E-16
**Th**	BP	GO:0045321~leukocyte activation	17	2.26E-15
**Th**	BP	GO:0030098~lymphocyte differentiation	14	3.74E-15
**Th**	BP	GO:0046649~lymphocyte activation	16	6.4E-15
**Th**	BP	GO:0001775~cell activation	17	1.4E-14
**Th**	BP	GO:0002521~leukocyte differentiation	14	6.8E-14
**Th**	BP	GO:0002520~immune system development	15	5.47E-11
**Th**	BP	GO:0030097~hemopoiesis	14	9.85E-11
**Th**	BP	GO:0048534~hemopoietic or lymphoid organ development	14	3.99E-10
**Ut**	BP	GO:0030326~embryonic limb morphogenesis	6	0.00000245
**Ut**	BP	GO:0035113~embryonic appendage morphogenesis	6	0.00000245
**Ut**	BP	GO:0035108~limb morphogenesis	6	0.00000567
**Ut**	BP	GO:0035107~appendage morphogenesis	6	0.00000567
**Ut**	BP	GO:0060173~limb development	6	0.00000671
**Ut**	BP	GO:0048736~appendage development	6	0.00000671
**Ut**	BP	GO:0051216~cartilage development	5	0.0000281
**Ut**	BP	GO:0060429~epithelium development	7	0.0000288
**Ut**	CC	GO:0031012~extracellular matrix	7	0.0000414
**Ut**	CC	GO:0005576~extracellular region	13	0.0000818
**Vg**	CC	GO:0005576~extracellular region	21	8.99E-14
**Vg**	BP	GO:0042628~mating plug formation	3	0.00000617
**Vg**	BP	GO:0045297~post-mating behavior	3	0.00000617
**Vg**	BP	GO:0007320~insemination	3	0.0000573
**Vg**	BP	GO:0007620~copulation	3	0.0000573
**Vg**	BP	GO:0019098~reproductive behavior	3	0.000347
**Vg**	BP	GO:0007618~mating	3	0.000347
**Vg**	CC	GO:0005615~extracellular space	6	0.00301
**Vg**	MF	GO:0008131~amine oxidase activity	2	0.0114
**Vg**	MF	GO:0048038~quinone binding	2	0.0128

Supplementary Table S4. Number of tissue-specific genes using different FC thresholds.

Tissues were sorted in descending order by the third column (FC≥4).

Tissue	Fold Change (FC)				
	2	4	8	16	32
**Te**	4051	2496	1635	1037	650
**Br**	1349	708	364	138	39
**Li**	421	280	208	156	107
**Sp**	557	223	76	23	3
**Fs**	347	208	143	79	38
**Th**	579	201	92	45	18
**Ki**	306	154	87	52	22
**Sin**	308	153	99	65	48
**Mu**	264	128	78	50	33
**Ov**	263	100	40	15	5
**Lu**	272	95	34	19	15
**Ag**	159	53	23	14	13
**Vg**	105	52	36	29	23
**Lin**	152	49	23	10	5
**Ut**	150	48	17	5	3
**He**	109	46	27	18	11
**St**	103	41	4	0	0

Supplementary Table S5. Expression profiles of 27 consistently and highly expressed genes. The "fpkm" column indicates mean fpkm value across all 17 tissues. "sd" and "se" stand for standard deviation and standard error respectively.

NO.	gene	fpkm	sd	se
**1**	Arf1	216.4004	71.5760	17.3597
**2**	Cox7a2l	157.9276	44.8052	10.8669
**3**	D8Ertd738e	87.8149	21.0976	5.1169
**4**	Eif1	328.4951	74.7391	18.1269
**5**	Eif4g2	271.2178	86.8054	21.0534
**6**	Eif5a	274.2647	93.5881	22.6984
**7**	Gabarap	246.6964	61.3683	14.8840
**8**	Grcc10	118.7790	34.1299	8.2777
**9**	Myeov2	137.9045	38.7013	9.3865
**10**	Ndufa2	163.5554	47.0850	11.4198
**11**	Ndufa7	158.1495	45.3430	10.9973
**12**	Nedd8	136.7558	36.2726	8.7974
**13**	Oaz1	251.1923	72.5944	17.6067
**14**	Pomp	144.5918	48.5043	11.7640
**15**	Psma2	117.5557	39.7861	9.6495
**16**	Psma3	162.6351	47.1668	11.4396
**17**	Psma4	113.0226	28.6832	6.9567
**18**	Psmb1	121.6363	28.7887	6.9823
**19**	Psmb2	87.5236	22.9753	5.5723
**20**	Psmb3	127.3378	36.7318	8.9088
**21**	Psmb4	137.0849	30.1077	7.3022
**22**	Psmb6	129.3216	33.1271	8.0345
**23**	Psmb7	142.6789	47.9245	11.6234
**24**	Rab1b	78.3410	13.1423	3.1875
**25**	Saraf	102.9171	30.4342	7.3814
**26**	Ubl5	184.7515	45.1253	10.9445
**27**	Vcp	111.2268	30.7782	7.4648
